# Molecular diversity and population structure at the Cytochrome P450 3A5 gene in Africa

**DOI:** 10.1186/1471-2156-14-34

**Published:** 2013-05-03

**Authors:** Ripudaman K Bains, Mirna Kovacevic, Christopher A Plaster, Ayele Tarekegn, Endashaw Bekele, Neil N Bradman, Mark G Thomas

**Affiliations:** 1 Research Department of Genetics, Evolution and Environment, University College London, Darwin Building, Gower Street, London, WC1E 6BT, UK; 2 Centre for Mathematics and Physics in the Life Sciences and Experimental Biology (CoMPLEX), University College London, Physics Building, Gower Street, London, WC1E 6BT, UK; 3 Addis Ababa University and Center of Human Genetic Diversity, P.O. Box 1176, Addis Ababa, Ethiopia; 4 Henry Stewart Group, 28/30 Little Russell Street, London, WC1A 2HN, UK; 5 Department of Evolutionary Biology, Uppsala University, Uppsala, Sweden

**Keywords:** Cytochrome P450 3A5, Africa, Population genetics, Gene-environment correlations, Pharmacogenetics

## Abstract

**Background:**

Cytochrome P450 3A5 (CYP3A5) is an enzyme involved in the metabolism of many therapeutic drugs. CYP3A5 expression levels vary between individuals and populations, and this contributes to adverse clinical outcomes. Variable expression is largely attributed to four alleles, CYP3A5*1 (expresser allele); CYP3A5*3 (rs776746), CYP3A5*6 (rs10264272) and CYP3A5*7 (rs41303343) (low/non-expresser alleles). Little is known about CYP3A5 variability in Africa, a region with considerable genetic diversity. Here we used a multi-disciplinary approach to characterize *CYP3A5* variation in geographically and ethnically diverse populations from in and around Africa, and infer the evolutionary processes that have shaped patterns of diversity in this gene. We genotyped 2538 individuals from 36 diverse populations in and around Africa for common low/non-expresser *CYP3A5* alleles, and re-sequenced the *CYP3A5* gene in five Ethiopian ethnic groups. We estimated the ages of low/non-expresser *CYP3A5* alleles using a linked microsatellite and assuming a step-wise mutation model of evolution. Finally, we examined a hypothesis that CYP3A5 is important in salt retention adaptation by performing correlations with ecological data relating to aridity for the present day, 10,000 and 50,000 years ago.

**Results:**

We estimate that ~43% of individuals within our African dataset express CYP3A5, which is lower than previous independent estimates for the region. We found significant intra-African variability in CYP3A5 expression phenotypes. Within Africa the highest frequencies of high-activity alleles were observed in equatorial and Niger-Congo speaking populations. Ethiopian allele frequencies were intermediate between those of other sub-Saharan African and non-African groups. Re-sequencing of *CYP3A5* identified few additional variants likely to affect CYP3A5 expression. We estimate the ages of CYP3A5*3 as ~76,400 years and CYP3A5*6 as ~218,400 years. Finally we report that global CYP3A5 expression levels correlated significantly with aridity measures for 10,000 [Spearmann’s Rho= −0.465, *p*=0.004] and 50,000 years ago [Spearmann’s Rho= −0.379, *p*=0.02].

**Conclusions:**

Significant intra-African diversity at the *CYP3A5* gene is likely to contribute to multiple pharmacogenetic profiles across the continent. Significant correlations between CYP3A5 expression phenotypes and aridity data are consistent with a hypothesis that the enzyme is important in salt-retention adaptation.

## Background

One of the main goals of the genomics revolution has been to characterize diversity within indigenous populations, which have traditionally been under-represented in research. The availability of genomic data is enabling researchers to identify how and why genomic variation affects individual and population differences in clinical outcomes following pharmaceutical drug administration. Additionally, evolutionary and demographic processes which have shaped population variation at clinically relevant regions of the human genome are now being determined. Studies of genes encoding drug metabolizing enzymes, such as the Cytochrome P450 (CYP450) super-family have identified variation which affects the safety and efficacy of therapeutic drugs. However little is known about intra-African variation at these loci. Africa is heavily burdened with common and infectious diseases [1], which are treated with multiple drugs. Studies of intra-African variation at genes encoding drug metabolizing enzymes are likely to be beneficial to clinicians, geneticists and researchers within the emerging field of evolutionary medicine [2]. They are also likely to have great potential for minimizing the risk of adverse clinical outcomes in patients with recent African ancestry [3].

CYP3A enzymes, a sub-family of the CYP450 super-family, are responsible for the phase I hepatic and intestinal metabolism of a wide spectrum of endogenous and xenobiotic compounds [4]. The two most clinically relevant CYP3A enzymes are CYP3A4 and CYP3A5, which together are involved in the metabolism of ~50% of all therapeutic drugs [5]. Because of the wide substrate range, some functional variation in *CYP3A* genes is associated with individual and population differences in pharmacogenetic profiles [6], adverse clinical outcomes [7], and elevated predisposition to diseases [8,9].

There is considerable inter-ethnic variability in CYP3A5 expression levels [10]. Individuals tend to express CYP3A5 at high concentrations (21-202 pmol/mg) or have significantly reduced, often undetectable, protein levels (<21 pmol/mg) [11-13]. Variability in protein expression is largely attributed to four *CYP3A5* alleles; CYP3A5*1, an expresser allele, and the low/non-expresser CYP3A5*3, CYP3A5*6 and CYP3A5*7 alleles [13,14]. Studies have reported that the highest frequencies of high-activity alleles are found in populations with recent African ancestry [15,16]. CYP3A5*3 is the main determinant of CYP3A5 expression levels in populations outside Africa [10]. The CYP3A5*6 and CYP3A5*7 alleles are observed almost exclusively in individuals with recent African ancestry [13-16], although CYP3A5*6 has been observed at low frequency in a sample of individuals from Los Angeles with Mexican ancestry, genotyped as part of the HapMap consortium. CYP3A5*7 has been observed at a frequency of 3% in ethnic Koreans [17]. There is some uncertainty over the functionality of the CYP3A5*6 mutation. Its effect on protein expression was reported in 2001 [13]. One of two cDNA products isolated from three CYP3A5*1/CYP3A5*6 heterozygotes did not contain the sequence for exon 7. Subsequent western blot analyses of liver samples from two CYP3A5*1/CYP3A5*6 heterozygotes found significantly lower protein levels than in CYP3A5*1 homozygotes. It has been proposed that CYP3A5*6 creates an aberrant splicing pathway [13], however this has not been confirmed experimentally. Although data presented by Kuehl *et al.* suggest that CYP3A5 expression levels in CYP3A5*6 carriers are lower than in CYP3A5*1 homozygotes, in the absence of expression analysis and more extensive *in vivo* and *in vitro* data we considered it prudent to allow for the possibility that in at least some individuals CYP3A5*6 is expressed. Unlike the CYP3A5*3 and CYP3A5*7 mutations, the association between CYP3A5*6 and clinical outcomes is not completely certain. A study examining the association between *CYP3A* genotypes and the metabolism of midazolam found a significant association between the metabolism of the drug and the presence of the CYP3A5*3 allele, but not the CYP3A5*6 allele [18]. However, an independent study of Japanese breast cancer patients found that tumor sizes were significantly higher in women who carried the CYP3A5*6 allele [19]. Given this uncertainty we present analyses that assume both that the CYP3A5*6 allele does, and does not affect protein expression and function.

A previous study reported that elevated CYP3A5*3 frequencies are positively correlated with increased geographic distance from the equator [20]. There is a latitudinal cline in the frequencies of alleles involved in heat adaptation, and consequently hypertension susceptibility [21]. A strong positive correlation is observed between latitude and functionally important variants of genes implicated in salt-sensitive hypertension, by regulating cardiovascular reactivity and volume avidity, such as angiotensinogen (*AGT*), G protein β3 subunit (*GNB3*), and epithelial sodium channel γ (*ENaCγ*) [21]. CYP3A5 is involved in the metabolism of renal cortisol to 6-β-hydroxycortisol, a key regulator of renal sodium transport, and immune responses which cause inflammation [22]. It has been proposed that the expresser CYP3A5*1 allele provides a selective advantage in equatorial populations due to the role of CYP3A5 in salt retention and the reabsorption of water [13,20]. Conversely, elevated CYP3A5*1 frequencies are hypothesized to be detrimental and are associated with elevated risk of salt-sensitive hypertension in non-equatorial populations [8,23,24]. The *CYP3A5* gene region has high frequencies of derived, functional alleles [25], and substantial population differentiation in the frequencies of the CYP3A5*3 allele when compared to neutral markers, as measured by weighted *F*_ST_ tests, [26]. This suggests that low/non-expression of CYP3A5 may be adaptive in non-equatorial populations.

Although CYP3A5 expression in Africa is likely to be highly variable, few previous studies have characterized intra-African diversity in *CYP3A5* and other clinically relevant genes. High levels of genetic diversity are observed within the continent compared to other geographic regions, and this is consistent with a recent African origin model of human evolution [27]. East Africa is a particularly diverse region of the continent. Reports have shown a gradual reduction in genetic diversity with increased geographic distance from Ethiopia [28-30] indicating that the region is one of the most genetically diverse in the world. Studies of functional variation in clinically relevant genes have found significant inter-ethnic differences within Ethiopia and between Ethiopian and other African populations [31-33]. These data highlight the potential that focused genetic studies of clinically relevant variation within Ethiopian populations have for understanding intra-African genetic diversity.

Within this study we have focused on characterizing *CYP3A5* variation in multiple geographically and ethnically diverse populations sampled from in and around Africa. We focused on determining population structure at this locus, and identified considerable population structuring within Africa. These results suggest that there are likely to be multiple pharmacogenetic profiles across Africa which could affect the safety and efficacy of many therapeutic drugs which are CYP3A5 substrates. Additionally, we report correlations between CYP3A5 expression phenotypes and aridity data for 10,000 and 50,000 years ago, consistent with a previous hypothesis that the enzyme is involved in salt retention/heat adaptation. This suggests that global variability in expression phenotypes may have occurred as a result of selective pressures on the gene.

## Results

### The prevalence of clinically relevant *CYP3A5* alleles in Africa

**Figure 1:**
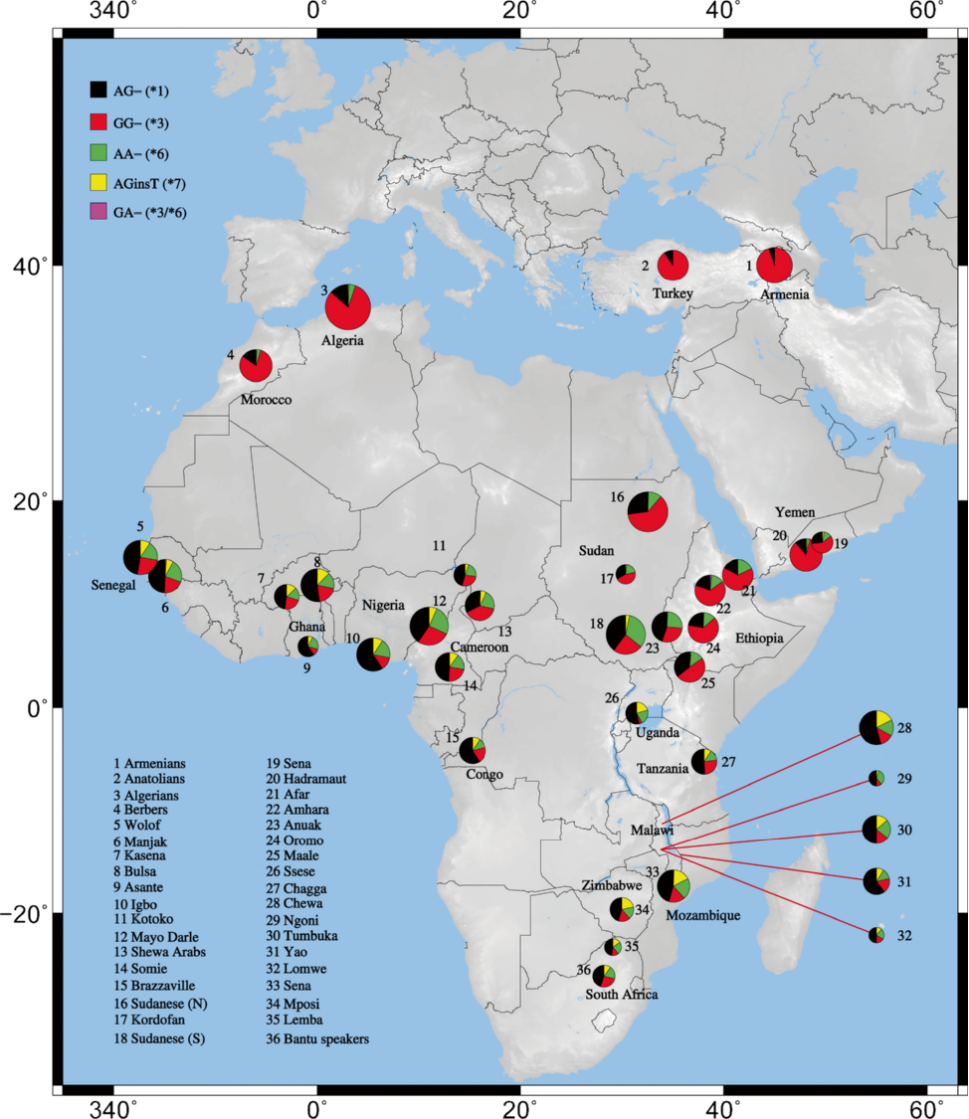
**The distribution of the five inferred *CYP3A5 *haplotypes across the dataset.** The size of each circle is proportional to the number of individuals sampled from a given population (see Additional file 1 Table S1). The allele combinations at all three loci are given in the key. The alleles in brackets define the inferred haplotype. N.B. the recombinant *CYP3A5* haplotype 5: *3/*6, is observed at low frequency in the dataset.

We genotyped 2245 individuals from 32 geographically and ethnically diverse African populations for common clinically relevant *CYP3A5* alleles. An additional 293 individuals from four non African populations from Europe and the Arabian Peninsula were also genotyped to permit comparisons of African diversity in a global context (Table 1). Prior to our study, the distribution of clinically relevant *CYP3A5* alleles across Africa, and relative to non-African populations, was unknown. We identified CYP3A5*1, CYP3A5*3 and CYP3A5*6 in all genotyped African population samples (allele frequency ranges: 4-81%, 4-81% and 4-33% respectively). CYP3A5*7 was confined almost exclusively to Niger-Congo speaking samples (range: 0-22%). The distribution of *CYP3A5* alleles is structured by major language family and geographic region, as evidenced by Analysis of Molecular Variance [*P<*0.0001 for both variables]. Pearson’s χ^2^ tests were carried out to examine within-region differences. Considerable heterogeneity was observed in East Africa [χ^2^=157.69, d.f.=21, *p*<0.0001] and North Africa [χ^2^=37.61, d.f.=9, *p*<0.01] but not in any other geographic region. The genotyped loci are in complete LD (*D´*=1, *p<*0.0001), except between the CYP3A5*6 and CYP3A5*1/*3 loci (*D´*=0.96, *p*<0.0001). A low frequency recombinant haplotype was observed in 10 heterozygotes explaining why *D*´ between CYP3A5*1 and CYP3A5*6 is not equal to 1. Haplotype analysis found that the low/non-expresser *CYP3A5* alleles occur predominantly on independent haplotype backgrounds (Figure 1 and Additional file 1 Table S1) suggesting that their convergent effects on CYP3A5 expression are independent. A significant correlation between pairwise genetic (*F*_ST_) and geographic distances (kilometers) was observed using a Mantel test when all populations genotyped in this study (*n*=36) were analyzed [Mantel *r* statistic=0.228, *p*<0.0001].

**Table 1:** **Genotype and allele frequencies and tests for deviation from Hardy-Weinberg Equilibrium (χ**^**2 **^***p*-values given)**

**Region**	**Country**	**Population**	CYP3A5*1/CYP3A5*3						CYP3A5*6						CYP3A5*7					
			**AA**	**AG**	**GG**	**Total**	G [*3]	**HWE**	**GG**	**GA**	**AA**	**Total**	A [*6]	**HWE**	**−/−**	**-/T**	**T/T**	**Total**	T [*7]	**HWE**
Europe	Armenia	Southern Armenians	0	10	90	100	0.95	1.00	100	0	0	100	0.00	N/A	100	0	0	100	0.00	N/A
	Turkey	Anatolian Turks	2	10	62	74	0.91	0.11	74	0	0	74	0.00	N/A	74	0	0	74	0.00	N/A
Arabian	Yemen	Yemeni from Hadramaut	2	21	59	82	0.85	1.00	77	5	0	82	0.03	1.00	80	2	0	82	0.01	1.00
Peninsula		Yemeni from Sena and Msila	7	17	13	37	0.58	0.74	29	7	1	37	0.12	0.42	35	2	0	37	0.03	1.00
North Africa	Algeria	Northern Algerians	9	42	108	159	0.81	0.12	146	15	0	161	0.05	1.00	159	2	0	161	0.01	1.00
	Morocco	Berbers	3	28	54	85	0.80	1.00	79	7	0	86	0.04	1.00	85	1	0	86	0.01	1.00
	Sudan	Northern Sudanese	24	58	51	133	0.60	0.29	104	28	0	132	0.11	0.36	135	1	0	136	0.00	1.00
		Sudanese from Kordofan	11	11	8	30	0.45	0.16	19	10	1	30	0.20	1.00	29	1	0	30	0.02	N/A
East Africa	Ethiopia	Afar	10	31	32	73	0.65	0.61	47	26	0	73	0.18	0.11	73	0	0	73	0.00	N/A
		Amhara	14	22	40	76	0.67	0.004	55	19	2	76	0.15	0.67	76	0	0	76	0.00	N/A
		Anuak	38	32	6	76	0.29	1.00	44	25	7	76	0.26	0.23	75	1	0	76	0.01	1.00
		Maale	20	36	19	75	0.49	0.82	53	22	0	75	0.15	0.34	74	1	0	75	0.01	1.00
		Oromo	12	28	34	74	0.65	0.20	55	19	1	75	0.14	1.00	75	0	0	75	0.00	N/A
	Republic of South Sudan	Southern Sudanese	74	42	9	125	0.24	0.46	58	50	15	123	0.33	0.42	117	8	0	125	0.03	1.00
	Tanzania	Chagga	28	18	4	50	0.26	0.71	36	14	0	50	0.14	0.57	41	9	0	50	0.09	1.00
	Uganda	Bantu speakers from Ssese	36	3	0	39	0.04	1.00	22	17	0	39	0.22	0.16	23	16	0	39	0.21	0.31
West Africa	Ghana	Asante	27	8	0	35	0.11	1.00	20	13	1	34	0.22	1.00	29	5	0	34	0.07	1.00
		Bulsa	58	29	3	90	0.19	1.00	61	28	0	89	0.16	0.11	69	19	2	90	0.13	0.62
		Kasena	28	17	2	47	0.22	1.00	31	16	0	47	0.17	0.32	35	12	0	47	0.13	1.00
	Senegal	Manjak	57	29	4	90	0.21	1.00	59	24	9	92	0.23	0.02	81	13	0	94	0.07	1.00
		Wolof	55	31	8	94	0.25	0.27	58	31	1	90	0.18	0.29	78	15	1	94	0.09	0.55
West Central	Cameroon	Kotoko	18	21	0	39	0.27	0.04	23	16	1	40	0.23	0.65	36	4	0	40	0.05	1.00
Africa		Shewa Arabs	26	31	12	69	0.40	0.62	42	24	3	69	0.22	1.00	60	9	0	69	0.07	1.00
		Mayo Darle	66	38	13	117	0.27	0.06	71	33	13	117	0.25	0.01	102	15	0	117	0.06	1.00
		Somie, Cameroonian Grassfields	36	28	1	65	0.23	0.16	44	19	2	65	0.18	1.00	52	13	0	65	0.10	1.00
	Congo	Congolese from Brazzaville	35	18	2	55	0.20	1.00	43	11	1	55	0.12	0.55	45	10	0	55	0.09	1.00
	Nigeria	Igbo	64	23	0	87	0.13	0.35	60	24	4	88	0.18	0.47	73	12	2	87	0.09	0.14
South East	Malawi	Chewa	66	25	1	92	0.15	1.00	66	23	3	92	0.16	0.69	60	31	0	91	0.17	0.06
Africa		Lomwe	13	4	1	18	0.17	N/A	10	8	0	18	0.22	N/A	14	4	0	18	0.11	N/A
		Ngoni	15	2	1	18	0.11	N/A	9	6	3	18	0.33	N/A	16	2	0	18	0.06	N/A
		Tumbuka	44	18	0	62	0.15	0.34	40	17	5	62	0.22	0.14	45	17	0	62	0.14	0.59
		Yao	37	18	1	56	0.18	0.67	43	12	1	56	0.13	1.00	46	10	0	56	0.09	1.00
	Mozambique	Sena	58	21	3	82	0.16	0.44	51	28	5	84	0.23	0.75	59	25	1	85	0.16	0.68
	South Africa	Bantu speakers	22	17	2	41	0.26	1.00	29	9	3	41	0.18	0.10	34	4	2	40	0.10	0.03
	Zimbabwe	Lemba	17	6	0	23	0.13	1.00	13	10	1	24	0.25	1.00	17	7	0	24	0.15	1.00
		Zimbabweans from Mposi	36	7	4	47	0.16	0.008	36	10	3	49	0.16	0.09	34	16	2	52	0.19	1.00

HWE could not be calculated for the Lomwe and Ngoni as both populations had fewer than 50 chromosomes meaning that the test had insufficient power. No population deviated from Hardy-Weinberg equilibrium, following Bonferonni correction (for CYP3A5*3: adjusted *p* value = 0.00139; correction for 36 tests, for CYP3A5*6: adjusted *p* value=0.0015; correction for 34 tests, for CYP3A5*7: adjusted *p* value=0.0017; correction for 30 tests). Deviations from HWE cannot be calculated for monomorphic loci: labeled “N/A” on the Table. “Total” refers to the number of individuals, from a given population, successfully genotyped at each locus. Population refers to the grouping of individuals either by self-declared ethnicity or geography/place collected.

The geographic and ethnic distributions of low-, intermediate- and high-expression phenotypes, based on haplotype frequencies were inferred. Expresser phenotypes were inferred assuming that CYP3A5*6 does and does not cause a low/non-expression phenotype (Additional file 2 Figure S1 and Additional file 3 Figure S2 respectively). The distributions in both Figures show that the highest frequencies of high-activity phenotypes are in equatorial regions of Africa, and Ethiopia has the highest within country inter-ethnic diversity, which is driven by differences between the Anuak and other Ethiopian groups.

### *Correlations between ecological variables and inferred* CYP3A5 *expression phenotypes*

A previous study reported a strong positive correlation between CYP3A5*3 allele frequencies and latitude [20]. Latitude is a correlate of multiple ecological variables that are associated with functional markers of genes involved in heat adaptation [21]. We tested for correlations between frequencies of low/non-expresser *CYP3A5* alleles, and inferred expresser phenotypes, with latitude and the ecological variables; temperature and precipitation (Table 2). Additionally, we tested for correlations with aridity indices calculated from temperature and precipitation data using the de Martonne aridity index [34]. This enabled us to consider the combined effect of temperature and precipitation on CYP3A5 phenotypes. Correlations were estimated using ecological data for the present day, and inferred for 10, 000 years ago (Holocene) and 50,000 years ago (Late Pleistocene) (http://badc.nerc.ac.uk/home/index.html). Correlations were performed assuming that CYP3A5*6 is a low/non-expresser allele, and that it is a neutral allele.

**Table 2:** **Correlation analyses, between ecological variables and *CYP3A5 *allelic and inferred expression data**

**Time period**	**Ecological variable**	***N=87***		***N*=36**											
		CYP3A5*3		CYP3A5*6		CYP3A5*7		High expresser allele (assuming CYP3A5*6 is a low/non-expresser allele)		Low expresser allele (assuming CYP3A5*6 is a low/non-expresser allele)		High expresser allele (assuming CYP3A5*6 is not a low/non-expresser allele)		Low expresser allele (assuming CYP3A5*6 is not a low/non-expresser allele)	
		**Rho**	***P*-value**	**Rho**	***P*-value**	**Rho**	***P*-value**	**Rho**	***P*-value**	**Rho**	***P*-value**	**Rho**	***P*-value**	**Rho**	***P*-value**
	**Latitude**	0.706	**<0.0001**	−0.331	**0.048**	−0.177	0.303	−0.472	**0.004**	0.472	**0.004**	−0.416	**0.012**	0.416	**0.012**
	**North latitude**	0.666	**<0.0001**	−0.621	**0.001**	−0.410	**0.047**	−0.659	**<0.0001**	0.659	**<0.0001**	−0.620	**0.001**	0.620	**0.001**
	**South latitude**	0.066	0.759	0.318	0.130	0.122	0.571	−0.701	**<0.0001**	0.701	**<0.0001**	−0.370	0.075	0.370	0.075
	**Temperature**	−0.664	**<0.0001**	0.268	0.114	0.494	**0.002**	0.655	**<0.0001**	−0.655	**<0.0001**	0.627	**<0.0001**	−0.627	**<0.0001**
**Present Day**	**Precipitation**	−0.129	0.232	−0.290	0.867	−0.150	0.384	−0.028	0.869	0.028	0.869	0.113	0.511	−0.113	0.511
	**Aridity**	0.286	**0.007**	−0.201	0.24	−0.267	0.116	−0.185	0.279	0.185	0.279	−0.029	0.868	0.029	0.868
	**Temperature**	−0.597	**<0.0001**	0.216	0.207	0.342	**0.041**	0.560	**0.0004**	−0.560	**0.0003**	0.635	**<0.0001**	−0.635	**<0.0001**
**Holocene**	**Precipitation**	0.072	0.510	−0.235	0.167	−0.522	**0.001**	−0.381	**0.022**	0.381	**0.022**	−0.190	0.266	0.190	0.266
	**Aridity**	0.471	**<0.0001**	−0.344	**0.04**	−0.575	**0.0002**	−0.465	**0.004**	0.465	**0.004**	−0.293	0.083	0.293	0.0832
	**Temperature**	−0.644	**<0.0001**	0.297	0.079	0.608	**<0.0001**	0.649	**<0.0001**	−0.649	**<0.0001**	0.641	**<0.0001**	−0.641	**<0.0001**
**Late Pleistocene**	**Precipitation**	0.160	0.139	−0.238	0.163	−0.353	**0.035**	−0.204	0.233	0.204	0.233	−0.023	0.892	0.023	0.892
	**Aridity**	0.532	**<0.0001**	−0.436	**0.008**	−0.480	**0.003**	−0.379	**0.026**	0.379	**0.023**	−0.211	0.216	0.211	0.216

For analyses with inferred CYP3A5 expression phenotypes high-, intermediate- and low- expression diplotypes were counted as genotypes and the frequencies of expresser and low/non-expresser alleles calculated. For analyses with phenotypes, CYP3A5*6 was considered to cause low/non-expression and to have no effect on CYP3A5 expression. Significant *p*-values, at the 5% level, are shown in bold. Rho indicates Spearmann’s Rho. “*N”* refers to the number of populations analyzed for each *CYP3A5* allele. For CYP3A5*3 frequencies, African data were combined with those previously reported [20]. For CYP3A5*6 and CYP3A5*7 correlations, only African data genotyped for this study were tested. North latitude and south latitude correlations were only performed with populations genotyped for this study.

Latitude correlated significantly with CYP3A5 expression in Africa [Spearmann’s Rho= −0.472, *p*=0.004], the correlation remained significant when considering north [Spearmann’s Rho= −0.659, *p*<0.0001] and south latitude [Spearmann’s Rho= −0.701, *p*<0.0001] populations separately. Across a global cohort (87 populations) which, included published genotyping data [20] and where CYP3A5*3 alone is considered to predict CYP3A5 expression levels, a significant correlation between latitude and frequencies of this allele was seen only for north latitude populations [Spearmann’s Rho= 0.666, *p*<0.0001], but not south [Spearmann’s Rho= 0.066, *p*=0.759]. No significant correlation was observed between aridity values for the present day and expresser phenotypes when CYP3A5*6 was considered a low/non expresser allele [Spearmann’s Rho= −0.185, *p*=0.279] or a neutral allele [Spearmann’s Rho= −0.0288, *p*=0.868]. Expresser phenotypes correlated significantly with aridity values from the Holocene [Spearmann’s Rho= −0.465, *p*=0.004] and Late Pleistocene [Spearmann’s Rho= −0.379, *p*=0.02] when CYP3A5*6 was considered as a low/non-expresser mutation. We subsequently examined independent correlations between expresser allele frequencies and temperature and precipitation. We found significant correlations between expresser allele frequencies and temperature for every time period, both when CYP3A5*6 was considered to be a low/non-expresser mutation and a neutral allele (*p*<0.0001 for every correlation, see Table 2). No significant correlation was observed between precipitation values and expresser allele frequencies.

We subsequently examined the correlations between present day ecological data and expresser allele frequencies, while controlling for geographic distances between populations, using partial Mantel tests. For each correlation CYP3A5*6 was assumed to be a low/non-expresser mutation. We found that the correlation between CYP3A5 expresser alleles and temperature remained significant when controlling for geographic proximity between populations [Mantel *r* statistic=0.398, *p*=0.02]. However the correlation with latitude was no longer significant [Mantel *r* statistic=0.202, *p*=0.05].

### *CYP3A5* variation observed in Ethiopia

Previous studies of genetic variation in drug metabolizing enzymes have identified considerable inter-ethnic diversity within Ethiopia and between Ethiopian and other African populations [31-33]. The results from our geographic survey of clinically relevant *CYP3A5* variants also indicated that there is considerable heterogeneity within Ethiopia, and between Ethiopia and other African populations. We performed a re-sequencing survey of the *CYP3A5* gene in five Ethiopian populations to characterize *CYP3A5* diversity in greater detail.

We observed significant inter-ethnic diversity in *CYP3A5* allele frequencies in Ethiopia. To identify additional variation and elucidate intra-Ethiopian population structure we re-sequenced an 8063bp region of *CYP3A5,* which included the *CYP3A5* promoter, exons and exon-flanking introns, in five Ethiopian populations. 51 polymorphic sites were identified (Table 3). Nine (17.6%) were exonic and, 3 out of 5 (6%) identified non-synonymous polymorphisms were predicted to adversely alter protein function. No significant difference in the proportion of synonymous or non-synonymous variation was identified by a codon-based Z-test [35] (*Z=*0.961 and *p=*0.169). The proportion of amino acid changes that we observed at the *CYP3A5* gene (5 changes/502 codons= ~1%) is higher than previously reported for 103 protein-coding genes (147 changes/26,999 codons=0.56%) [36], although the differences are not significant [paired *t* test, *t*=1.01, d.f.=1, *p*=0.50]. We did not identify any variants in experimentally established transcription factor binding sites [37,38]. Eight of the nine identified promoter variants occurred in nucleotide positions that are highly conserved in primates (i.e. where the allele is the same in all primate species), and bioinformatic analyses predicted that four out of nine may affect transcription factor binding. Of all identified polymorphisms – predicted and previously reported to affect CYP3A5 expression and activity (*n=*10) – 4 (2 promoter, CYP3A5*3 and CYP3A5*6) occurred at frequencies over 1%. The highest frequency variants identified were CYP3A5*3, CYP3A5*6 and the non-functional variant rs15524, which is found in high LD with CYP3A5*3[39].

**Table 3:** **All polymorphic sites identified in an 8063bp *CYP3A5 *region re-sequenced in five Ethiopian populations**

					**Afar**		**Amhara**		**Anuak**		**Maale**		**Oromo**		**Total**	
**Region of *CYP3A5***	**Position on chromosome 7**	**Position relative to the translation initiation codon (A of ATG is +1)**	**dbSNP database refSNP ID**	**Effect**	***f***	**n**	***f***	**n**	***f***	**n**	***f***	**n**	***f***	**n**	***f***	**n**
**Promoter**	99278314	−795 T>A	rs3823812		0.00	3	0.00	3	0.01	4	0.01	10	0.01	5	0.0331	25
**Promoter**	99278267	−748 C>G			0.01	5	0.00	2	0.00	1	0.00	1	0.01	6	0.0198	15
**Promoter**	99278224	−705 3 base pair deletion			0.00	1	0.00	1	0.01	5	0.00	1	0.00	3	0.0146	11
**Promoter**	99278223	−704 A>G			0.00	0	0.00	0	0.00	0	0.00	1	0.00	0	0.0013	1
**Promoter**	99278152	−633 C>A			0.00	0	0.00	0	0.00	0	0.00	0	0.00	1	0.0013	1
**Promoter**	99278146	−627 G>A			0.00	0	0.00	0	0.00	1	0.00	0	0.00	0	0.0013	1
**Promoter**	99278144	−625 A>G			0.00	0	0.00	0	0.00	0	0.00	1	0.00	0	0.0013	1
**Promoter**	99278070	−551 C>A	rs28365079		0.01	4	0.01	5	0.02	15	0.01	8	0.01	4	0.0476	36
**Promoter**	99277988	−469 G>A			0.00	0	0.00	0	0.00	0	0.00	1	0.00	0	0.0013	1
**UTR of exon 1**	99277593	−74 C>T	rs28371764		0.00	2	0.01	6	0.00	0	0.00	2	0.00	2	0.0158	12
**UTR of exon 1**	99277544	−25 A>C			0.00	0	0.00	0	0.00	0	0.00	0	0.00	1	0.0013	1
**Intron 1**	99277392	127 G>A			0.00	0	0.00	0	0.00	1	0.00	2	0.00	0	0.0040	3
**Intron 1**	99277337	182 C>A			0.00	0	0.00	0	0.00	3	0.00	0	0.00	0	0.0040	3
**Intron 2**	99272310	5209 C>T	rs28365067		0.01	11	0.02	12	0.01	5	0.01	8	0.01	8	0.0580	44
**Intron 2**	99272290	5229 G>A	rs41301652		0.00	0	0.00	0	0.00	2	0.00	0	0.00	0	0.0026	2
**Intron 2**	99272275	5244 C>T			0.00	0	0.00	0	0.00	0	0.00	0	0.00	2	0.0026	2
**Intron 3**	99272103	5416 C>T			0.00	0	0.00	0	0.00	0	0.00	0	0.00	2	0.0026	2
**Intron 3**	99272009	5510 T>A	rs28969392		0.01	6	0.01	4	0.01	10	0.01	9	0.00	3	0.0422	32
**Intron 3**	99271928	5591 C>T	rs41301655		0.00	0	0.01	4	0.00	1	0.00	0	0.00	2	0.0092	7
**Intron 3**	99271853	5666 A>G	rs41301658		0.00	1	0.00	1	0.00	3	0.01	7	0.00	2	0.0185	14
**Intron 3**	99271808	5711 A>G	rs41258334		0.01	11	0.01	11	0.01	5	0.01	9	0.01	8	0.0580	44
**Intron 3**	99271778	5741 A>G			0.01	6	0.00	3	0.01	4	0.01	8	0.00	3	0.0317	24
**Intron 3**	99270539	6980 A>G	rs776746	Defines the variant CYP3A5*3	0.13	95	0.14	102	0.06	44	0.10	75	0.13	97	0.5581	413
**Intron 3**	99270504	7015 3 base pair deletion			0.00	0	0.00	0	0.00	0	0.00	1	0.00	0	0.0014	1
**Intron 3**	99270318	7201 C>T	rs8175345		0.00	0	0.00	1	0.01	9	0.00	0	0.00	1	0.0149	11
**Exon 4**	99270249	7270 G>A		G77S	0.00	0	0.00	0	0.00	0	0.00	1	0.00	0	0.0014	1
**Intron 4**	99270164	7355 C>T	rs28365074		0.00	0	0.00	0	0.00	1	0.00	0	0.00	2	0.0041	3
**Intron 5**	99264352	13167 T>C	rs68178885		0.00	3	0.00	2	0.00	1	0.00	3	0.00	1	0.0132	10
**Intron 6**	99264149	13370 G>A	rs41301670		0.00	0	0.00	0	0.00	0	0.00	0	0.00	2	0.0027	2
**Exon 7**	99262835	14684 G>A	rs10264272	Defines the variant CYP3A5*6	0.04	28	0.03	23	0.05	39	0.03	23	0.03	21	0.1763	134
**Exon 7**	99262793	14726 A>G	rs2838372	Synonymous	0.00	1	0.00	0	0.00	0	0.00	0	0.00	0	0.0013	1
**Intron 7**	99262642	14877 A>G			0.00	1	0.01	5	0.02	12	0.01	9	0.00	2	0.0382	29
**Intron 7**	99261737	15782 T>C	rs28969393		0.01	5	0.01	4	0.01	9	0.01	9	0.00	3	0.0396	30
**Exon 8**	99261651	15868 A>G		K266R	0.00	0	0.00	0	0.00	1	0.00	0	0.00	0	0.0013	1
**Intron 8**	99261583	15936 C>A			0.00	0	0.00	0	0.00	0	0.00	2	0.00	0	0.0026	2
**Intron 8**	99260546	16973 G>A			0.00	0	0.00	1	0.00	0	0.00	0	0.00	0	0.0013	1
**Exon 9**	99260502	17017 C>T		R268Stop	0.00	0	0.00	0	0.00	1	0.00	0	0.00	0	0.0013	1
**Intron 9**	99260407	17112 C>T	rs28383478		0.00	0	0.00	2	0.00	0	0.00	0	0.00	0	0.0026	2
**Intron 9**	99260362	17157 G>T	rs4646453		0.00	3	0.00	3	0.01	4	0.01	10	0.01	5	0.0331	25
**Intron 9**	99260282	17237 T>G			0.00	0	0.00	0	0.00	1	0.00	0	0.00	0	0.0013	1
**Intron 9**	99260170	17349 T>G			0.00	3	0.00	2	0.01	7	0.01	7	0.00	3	0.0291	22
**Intron 9**	99258524	18995 C>T	rs10247580		0.00	0	0.00	2	0.02	12	0.01	7	0.00	1	0.0291	22
**Intron 9**	99258320	19199 G>A			0.00	0	0.00	0	0.00	1	0.00	0	0.00	0	0.0013	1
**Intron 9**	99258316	19203 T>C			0.00	0	0.00	0	0.00	0	0.00	2	0.00	0	0.0026	2
**Exon 10**	99258124	19395 A>C		K342T	0.00	0	0.00	0	0.00	0	0.00	0	0.00	1	0.0013	1
**Exon 11**	99250397	27125-27126 T insertion	rs41303343	Defines the variant CYP3A5*7	0.00	0	0.00	0	0.00	1	0.00	1	0.00	0	0.0026	2
**Exon 11**	99250381	27138 A>G		V350M	0.00	0	0.00	0	0.00	1	0.00	0	0.00	0	0.0013	1
**Intron 12**	99247647	29872 G>T			0.00	0	0.00	0	0.00	0	0.00	2	0.00	0	0.0026	2
**Intron 12**	99247503	30016 1 base pair deletion	rs28365093		0.00	3	0.01	4	0.02	15	0.01	8	0.01	4	0.0450	34
**Intron 12**	99246026	31493 T>C	rs28365069		0.01	4	0.01	11	0.01	11	0.02	18	0.01	9	0.0699	53
**3' UTR**	99245914	31605 C>T	rs15524		0.14	105	0.14	109	0.09	69	0.11	84	0.14	107	0.6253	474

*n* refers to the total number of chromosomes on which a particular variant was observed. *f* is the relative frequency of each variant. Total refers to the number of times a variant was observed in the Ethiopian cohort (758 chromosomes) and *f* is its relative frequency. Position on chromosome 7 is based on NCBI Build 132, February 2009.

### Ethiopian *CYP3A5* variation in the context of other geographic populations

We analyzed the Ethiopian re-sequencing data along with those previously reported for three ethnically diverse populations from the Corriell Repositories to analyze the data in a global context [20] (Table 4). The results of the Hudson-Kreitman-Aguadé (HKA) test [40], comparing intra- and inter-species *CYP3A5* diversity, was not significant (*p=*0.6346). Tajima’s *D*, Li’s D* and F*, Fu and Li’s *F* and *D* (using chimpanzee sequence to establish ancestral states), and Fu’s *F*_*S*_ all indicated a skew towards rare variants in every population, which is consistent with general human population growth or positive selection. Fu and Li’s D* and F* reported a significant departure from neutrality for both Europeans and the Anuak, although significance was only reached for Europeans following Bonferonni correction (8 tests). Fu and Li’s *F*_*S*_ reported a significant departure from neutrality for 7 of the 8 populations after Bonferonni correction. Strobeck’s *S* results were consistent with Fu’s *F*_*S*_*,* as expected. The results of the *H* test, used to assess whether there is an excess of high frequency derived variants [41], were not significant in any population (*p>*0.05), however nucleotide diversity at *CYP3A5* is low and this may be affecting the tests.

**Table 4:** **A summary of the tests for departures from neutrality for an 8063bp region of *CYP3A5***

	**Global populations**			**Ethiopian populations**				
	**African-Americans**	**Europeans**	**Han Chinese**	**Afar**	**Amhara**	**Anuak**	**Maale**	**Oromo**
**Sample size**	23	24	23	75	76	76	76	76
**Nucleotide diversity (π)**	5.4 x 10^-4^	9 x 10^-5^	1.1 x 10^-4^	2.1 x 10^-4^	2.5 x 10^-4^	3.6 x 10^-4^	3.5 x 10^-4^	2.2 x 10^-4^
**McDonald-Kreitman test**	0.475	0.50	0.777	0.462	0.475	0.576	1.00	0.777
**Tajima’s *D***	−1.04	−1.92	−1.21	−1.46	−1.13	−1.26	−0.96	−1.79
Fu and Li’s D*	−0.97	**−2.86**	−1.82	−1.19	0.12	−2.72	−0.11	−1.05
Fu and Li’s F*	−1.17	**−3.00**	−1.91	−1.54	−0.43	−2.57	−0.53	−1.58
**Fu and Li’s *D***	−0.64	−1.37	−1.45	−1.56	−0.08	−1.93	0.73	−0.96
**Fu and Li’s *F***	−0.92	−1.81	−1.55	−1.79	−0.52	−1.96	0.13	−1.48
**Fu’s *F***_***S***_	**−31.06**	**−5.71**	−1.54	**−9.48**	**−11.74**	**−18.44**	**−11.08**	**−22.84**
**Strobeck’s *S***	**1.00**	**0.999**	0.929	**1.00**	**1.00**	**1.00**	**1.00**	**1.00**
**Fay and Wu’s *H *statistic**	−0.13140	−3.25532	−1.89372	0.10774	−0.23998	−0.47177	−0.87086	−1.75514

Statistically significant departures from neutrality, following Bonferonni correction (correction for 8 tests; adjusted *p*≤0.00625) are shown in bold.

**Table 5:** **Pairwise *F***_**ST **_**values for five Ethiopian populations and three other global populations**

	**Afar**	**Amhara**	**Anuak**	**Maale**	**Oromo**	**African-Americans**	**Europeans**	**Han Chinese**
**Afar**	*	0.74597	**<0.00001**	**0.00436**	0.76537	**<0.00001**	**<0.00001**	**<0.00001**
**Amhara**	−0.00248	*	**<0.00001**	**0.00347**	0.93525	**<0.00001**	**<0.00001**	**<0.00001**
**Anuak**	0.04566	0.05138	*	**0.00257**	**<0.00001**	**<0.00001**	**<0.00001**	**<0.00001**
**Maale**	0.01951	0.01736	0.01061	*	**0.00267**	**<0.00001**	**<0.00001**	**<0.00001**
**Oromo**	−0.00255	−0.0036	0.04981	0.01547	*	**<0.00001**	**<0.00001**	**<0.00001**
**African-Americans**	0.08997	0.09366	0.01558	0.03432	0.08803	*	**<0.00001**	**<0.00001**
**Europeans**	0.04873	0.03807	0.15448	0.08716	0.03371	0.19028	*	**0.00257**
**Han Chinese**	0.10812	0.0893	0.23763	0.16215	0.09715	0.29154	0.0677	*

Pairwise *F*_ST_ values are shown in the bottom left side of the Table, the corresponding *p-*values are shown in the top right of the Table. *P*-values which are significant after Bonferroni correction (adjusted *p*-value = 0.00625; correction for 8 tests) are shown in bold.

**Table 6:** **Age estimates of clinically relevant *CYP3A5 *variants**

***CYP3A5 *variant**	**Location on chromosome 7**	**Location in gene**	**Allele dated**	**Number of chromosomes**	**Average squared distance (ASD)**	**Time to most recent common ancestor**		**95% confidence intervals of allele age estimate based on a star phylogeny**			
						**Estimate of allele age**		**Lower**		**Upper**	
						**Generations**	**Years**	**Generations**	**Years**	**Generations**	**Years**
CYP3A5*3	99270539	Intron 3	G	134	1.0746	2388	76,416	1797	57,504	3211	102,752
CYP3A5*6	99262835	Exon 7	A	18	3.0714	6825	218,400	3086	98.752	11975	383,200
**rs15524**	99245914	3´ UTR	T	324	1.8426	4095	131,040	3157	101,024	5413	173,216

Allele ages were estimated using a mutation rate of 0.00045 and a generation time of 32 years. The confidence intervals for the estimated age of the CYP3A5*6 are large; most likely a reflection of the small sample size. UTR is the Untranslated Region.

**Figure 2:**
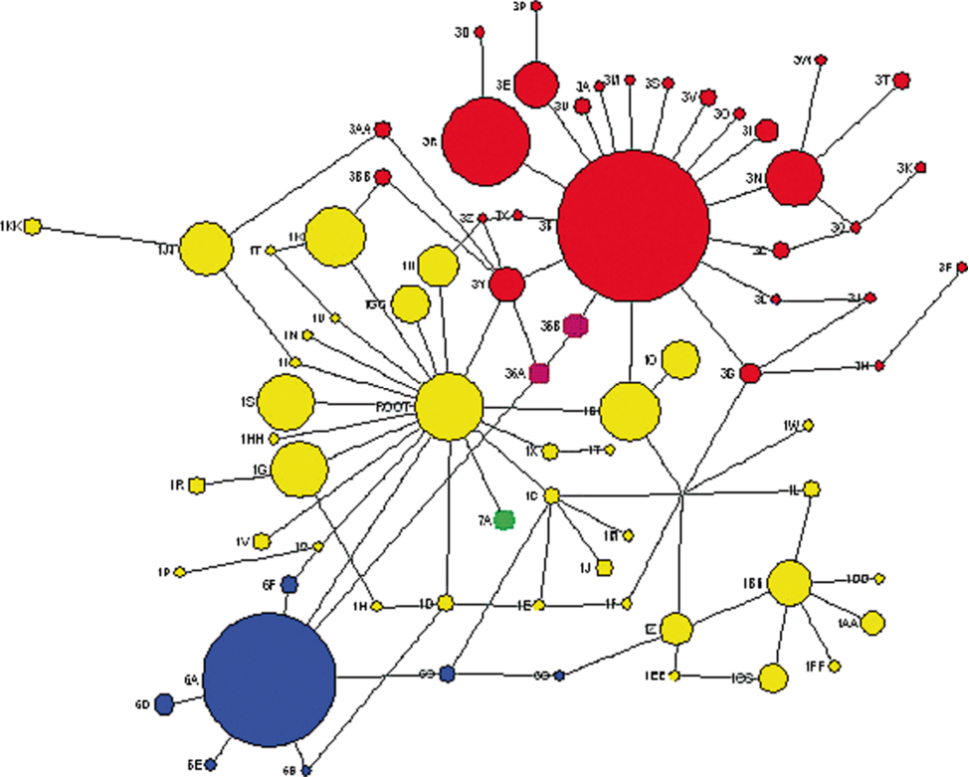
**A network of all *CYP3A5 *haplotypes inferred for Ethiopians and Coriell populations.** Networks assume single mutational steps. Haplotypes are colored according to the haplogroup to which they belong; haplotypes which are defined by the CYP3A5*3 allele are shown in red; those defined by CYP3A5*1 in yellow; CYP3A5*7 in green; CYP3A5*6 in blue and CYP3A5*3/*6 recombinant haplotypes are shown in purple. The size of each haplotype is proportional to its frequency in the global database. Additional file 4 Figure S3a contains information on the exact composition of each coded haplotype.

72 haplotypes were inferred from allelic data for all 8 Ethiopian population samples, 33 (45.8%) containing CYP3A5*1, 29 (40.3%) containing CYP3A5*3, 7 (9.7%) containing CYP3A5*6, 1 (1.4%) containing CYP3A5*7, and 2 (2.8%) containing both CYP3A5*3 and CYP3A5*6 (Additional file 4 Figures S3a and b). LD across the gene is high. A phylogeny, based on network analysis of the haplotype data, is presented in Figure 2. 98% of European and 83% of Han Chinese haplotypes contain the CYP3A5*3 allele, as do ~64% of Afar haplotypes and ~67% in both the Amhara and Oromo. Gene diversity is highest in African Americans (0.963 ± 0.02) and lowest in Europeans (0.589 ± 0.08). The CYP3A5*1 haplogroup is significantly more diverse than the other haplogroups (0.921 ± 0.01) (*p<*0.0001 for every comparison). Population differentiation was measured by pairwise *F*_ST_ (Table 5). The Afar, Amhara and Oromo are intermediate between individuals with recent African ancestry and Han Chinese and European groups. We placed population structure seen at the *CYP3A5* gene in a wider genomic context by analyzing intra-Ethiopian differentiation at markers on the non-recombining regions of the Y chromosome (NRY) and the mitochondrial genome (hypervariable region 1 [HVS1] and coding region SNPs) [42]. We compared Ethiopian NRY and HVS1 genotypes with data for 92 Fars from Iran, 95 Nigerian Igbo, 126 Greek-Cypriots and 60 Halfawi from the Republic of Sudan. The Anuak are outliers compared to the other Ethiopian populations (data not shown), consistent with genome wide-markers [43] and what we report for *CYP3A5*. Intra-Ethiopian population structure at the *CYP3A5* gene is also consistent with that seen at other drug metabolizing genes *CYP1A2*[31], and *UGT1A1*[32].

### Estimating the age of clinically relevant *CYP3A5* alleles

The age of an allele is the time since it arose by mutation [44,45]. Estimating the ages of *CYP3A5* alleles may help to identify specific demographic processes which have affected inter-population differences in allele frequencies, or identify an important role for natural selection in selecting for specific alleles [44]. Under the stepwise mutation model of microsatellite evolution, and assuming no recombination, we estimated the time to the most recent ancestor (TMRCA) of the CYP3A5*3 mutation to be 2388 generations (95% confidence intervals [C.I.]: 1797–3211) and CYP3A5*6 to be 6825 generations (95% C.I.: 3086–11,975). Assuming that a generation is 32 years [46], the estimated age of the CYP3A5*3 mutation is ~76,416 years (95% C.I. 57,504-102,752 years) and CYP3A5*6 is 218,400 years (95% C.I. 98,752-383,200 years) (Table 6). Our estimates of the age of CYP3A5*3 is consistent with its presence within and outside of Africa. The distribution of the CYP3A5*6 allele shows some similarity to that of FMO2*1, an allele of the gene encoding the drug metabolizing enzyme FMO2 [33]. FM02*1 occurs at similar frequencies across Africa and is not found at high frequencies outside of the continent. The estimated age of FMO2*1 is 502,404 years (95% C.I. 154,790–1,041,243 years) based on a coalescent simulation [47] and using data from populations re-sequenced as part of the NIEHS SNPs database (http://egp.gs.washington.edu/). The age estimates of both the CYP3A5*6 and FMO2*1 alleles predate estimates of the range-expansion of modern humans out of Africa.

## Discussion

We performed an extensive geographic survey of clinically relevant *CYP3A5* alleles in a large African cohort and found highly variable frequencies of the ancestral CYP3A5*1 allele (9-96%) across the continent. We estimate that ~43% of individuals within our African dataset express CYP3A5, which is much lower than all other previous estimates for the continent (between 55-95%) [15,16]. The classification of *CYP3A5* alleles as expresser or low/non-expresser will affect estimates of expresser frequencies in Africa. *In vitro* studies of CYP3A5 expression levels in CYP3A5*6 homozygotes are needed to establish the effect of the mutation on protein expression. The results from such studies may alter the classification of CYP3A5*6 as a clinically relevant *CYP3A5* allele, and mean that CYP3A5 protein expression levels across Africa are likely to be consistent with those presented in Additional file 3 Figure S2 . Our estimates of the proportion of CYP3A5 expressers differ across Africa, consistent with the Sahara acting as a barrier to gene flow [48,49]. Additionally, we estimate that the proportion of CYP3A5 expressers in East Africa (~36%) is lower than in other regions of sub-Saharan Africa (~45%), and report considerable heterogeneity among Ethiopian ethnic groups (17-54%). We found that the highest frequencies of inferred high-activity phenotypes were seen in equatorial and Niger-Congo speaking populations.

From the geographic survey we observed that the Ethiopian allele frequencies are intermediate between sub-Saharan African and Eurasian groups [50]. Our study has extended previous work on *CYP3A5* in Ethiopia [51] by accounting for, and identifying, considerable inter-ethnic variability within the country. *CYP3A5* haplotype diversity and structure in the Afar, Amhara and Oromo were characteristic of that seen in European Caucasians and Han Chinese individuals. There is a known Arabian contribution to Ethiopian ancestry as a result of migration of Semitic groups into the region, which has influenced genetic diversity [48,52]. We further examined intra-Ethiopian diversity at mitochondrial and Y-chromosome genetic markers and found that the Anuak were outliers. This suggests that the intra-Ethiopian diversity we observed can be explained by Arabian admixture in the Afar, Amhara and Oromo, rather than differential selection pressures on *CYP3A5*.

Considerable intra-African population structuring at the *CYP3A5* gene suggests that there are likely to be multiple pharmacogenetics profiles for key drugs used across the continent, including many used in the treatment and control of malaria [53] and HIV-1 [54]. We identified significant differences between Ethiopians and other sub-Saharan African populations, and intra-Ethiopian diversity, at the *CYP3A5* gene. The results from our study suggest that East Africans are likely to be distinct from a wider cohort of African patients, and that there are likely to be inter-ethnic differences within East Africa. The results from large surveys [32,33], including our study, emphasize the importance of including sub-Saharan African populations in pharmacogenetics research; over 90% of the global disease burden is found in developing countries [55,56]. An appreciable number of the diseases found within the region are treated with CYP3A5 substrates [5,57] at doses optimized for patients with recent European ancestry [26]. Larger and more detailed surveys of clinically important variation in diverse African populations will improve our understanding of how specific drugs and dosages contribute to adverse clinical outcomes within Africa and the African Diaspora. The number of such studies will undoubtedly increase with the availability of newer and cheaper sequencing technologies [58,59] and progression towards the $1000 genome [60].

We combined our African CYP3A5*3 data with those previously published to examine the global prevalence of the allele. We found a significant, positive correlation between CYP3A5*3 allele frequencies and latitude, consistent with a previous report [20]. This correlation remained significant when only African data were considered [Spearmann Rho= 0.666, *p*<0.0001]. In contrast we found no significant correlation between latitude and CYP3A5*6 or CYP3A5*7 frequencies. Given the restricted geographic distribution of the CYP3A5*6 allele mainly to Africa, coupled with our estimates of its age (>200,000 years), it is possible that this allele was lost in a population bottleneck during the range-expansion of humans out of Africa. The heterogeneous distribution of CYP3A5*7 in Africa suggests that it arose from a much more recent mutation event and may have spread with the expansion of Niger-Congo speaking populations ~4000 years ago [61]. Nonetheless, the reasons why the derived CYP3A5*3, CYP3A5*6 and CYP3A5*7 alleles are found at appreciable frequencies in sub-Saharan Africa remains unknown, and the possibility of independent evolutionary causes cannot be discounted. The global distribution of the CYP3A5*3 allele is unusual when compared with microsatellite markers, genotyped in samples from the Human Genome Diversity Panel (HDGP-CEPH) [20]. Integrated haplotype scores (iHS) for CYP3A5*3 haplotypes in HGDP-CEPH populations sampled from high latitudes north and south of the equator are outliers in the iHS genome-wide distribution (iHS≥2) [62]. iHS scores of CYP3A5*3 haplotypes are similar to those of genomic regions surrounding the *LCT* (lactase) and *CD36* genes [63], which have both been reported to have undergone positive selection [64-66]. It is plausible that an increase in latitude of ~20°, when humans first expanded from East Africa to the Arabian Peninsula, is coupled with specific environmental changes which provided a novel selection pressure. Temperature and precipitation data associated with the Quaternary QUEST project (accessed through the British Atmospheric Centre: http://badc.nerc.ac.uk/home/index.html) suggest that changes in precipitation over the past 50,000 years are greater than those in temperature. However we did not find any significant correlation between precipitation values and *CYP3A5* allele frequencies. We did observe negative correlations between inferred expression phenotypes (assuming CYP3A5*6 is a low/non-expresser allele) and aridity values for the Holocene [Spearmann’s Rho= −0.465, *p*=0.004] and Late Pleistocene [Spearmann’s Rho= −0.379, *p*=0.02]. Under the de Martonne aridity index, this means that high frequencies of high-activity alleles are positively correlated with arid and semi-arid environments [34]. This finding is consistent with the hypothesis that high-activity *CYP3A5* alleles may be adaptive in regions where there are frequent water shortages, by aiding the rapid retention of water [20]. However, stronger correlations were found with temperature alone. Although further work will be needed to confirm these ecological correlations, the strong correlation with temperature is consistent with what we would expect for functional variation of genes involved in heat adaptation [21]. However, we cannot rule out that there may be an, as yet untested, ecological variable which may have provided a selective pressure.

We have provided the first estimate of the age of the CYP3A5*3 allele as ~76,000 years (95% C.I. 57,504-102,752 years), using a closely linked microsatellite. This estimate is consistent with the wide geographic distribution of CYP3A5*3, both inside and outside Africa although not with the low haplotype diversity [20,67] and high iHS scores [62] previously reported. This may be because the allele age estimation presented here was made using Ethiopian data whereas the previously reported low haplotype diversity [20,67] and high iHS scores [62] were based on non-African samples. It is also possible that the microsatellite mutation rate for rs10536492 differs from the genome wide average for dinucleotide repeats [68], which would influence the age estimates, or that the extensive LD on haplotypes containing CYP3A5*3 is explained by unusually low recombination rates in that genomic region. Finally, while the correlations between the CYP3A5*3 geographic distribution and ecological variables relating to temperature and aridity, as well as previously reported low haplotype diversity [20,67] and high iHS scores [62], both independently support the hypothesis that the CYP3A5*3 allele is adaptive, alternative targets of positive selection on the same haplotype background may exist and both they (if present) and the CYP3A5*3 allele may have been differentially selected inside and outside Africa.

## Conclusions

The data that we present complement and extend work in previous publications which reported evidence of geographically restricted positive selection on the *CYP3A5* gene [20,25,26,69]. In addition to improved knowledge of the effect and distribution of clinically relevant genetic variation, our approach highlights the importance of considering population history and of utilizing evolutionary approaches in clinical research. Evolutionary approaches to genetic studies are likely to identify additional populations that require targeted health interventions. Further studies which characterize variation in medically important genes in ethnically and geographically diverse global populations are needed as we progress towards personalized clinical medicine, a key goal of the genomics revolution [70].

## Methods

### Samples

The DNA samples analyzed in this study were part of a collection at The Centre for Genetic Anthropology at University College London. Samples were collected anonymously and with informed consent (verbal in Africa) from ostensibly healthy individuals, between 1998–2007, from specified locations in and around Africa [ethical approval: UCLH 99/0196]. Additional ethical approval was obtained for Ethiopian collections from the National Health Research Ethical Clearance Committee under the Ethiopian Science and Technology Commission in Addis Ababa. All samples have been previously used in studies on clinically relevant genes [31-33]. For analyses, individuals were grouped by the collection location or by ethnicity (Additional file 5 Table S2). Samples were not grouped according to country as the partitioning of much of the African continent by colonial powers was recent and largely irrespective of ethnic identities [71]. 1028 CYP3A5*1/*3 genotypes for 51 global populations, from the Human Genome Diversity Panel-Centre d’Etude du Polymorphisme Humain (HGDP-CEPH) collection, which had previously been published [20] were combined with the 2538 sample cohort genotyped for this study. *CYP3A5* re-sequencing data, which were previously published, for 70 individuals from three distinct ethnic groups from the Coriell Repositories (24 European Caucasians, 23 African-Americans and 23 Han Chinese individuals) were combined with the Ethiopian cohort for detailed integrative analyses [20]. Published data were provided by Dr Emma Thompson from the University of Chicago.

### Genotyping and re-sequencing

*Genotyping of clinically relevant CYP3A5 alleles;* Genotyping of CYP3A5*1, CYP3A5*3, CYP3A5*6 and CYP3A5*7 was performed using TaqMAN allelic discrimination technology [ABiosystems product code: C_26201809_30 for CYP3A5*1/*3, and ABiosystems product code: C_30203959 for CYP3A5*6], and KASPar (performed externally by KBiosciences®, UK).

*Re-sequencing of CYP3A5;* The 13 exons and their flanking introns, promoter region and 3´ untranslated of *CYP3A5* were amplified in 379 Ethiopian individuals using primers designed on the basis of the *CYP3A5* reference sequence in NCBI Build 132 [(http://www.ncbi.nlm.nih.gov/) (see Additional file 6 Table S3 for a list of primers)]. Amplicons were sequenced using ABI PRISM Dye Terminators version 3.1 on an ABI 96-capillary 3730×l DNA Analyzer according to the manufacturer’s protocol (Applied Biosystems, Applera, UK). Part of the *CYP3A5* gene was re-sequenced externally by Macrogen®, USA.

*Microsatellite genotyping;* A –GT microsatellite, located ~1500 base pairs downstream of the 3´ end of *CYP3A5* was genotyped in 379 Ethiopian individuals, for whom re-sequencing data were also generated. Microsatellite genotyping was performed using a high-throughput method adapted from [72]. A 456 base pair region of *CYP3A5,* approximately ~1000 base pairs downstream of the 3´ UTR was amplified using the forward primer 5´-AATATATGTGTTTGTATGTGTG-3´ and a fluorescently labeled reverse primer FAM-AAGTGCTACCAATTTTGTACGT-3´. PCR amplification was performed in 10 μl reaction volumes containing 1ng of template DNA, 0.5 μM of primers, 0.2 units *Taq* DNA polymerase (HT Biotech, Cambridge, UK), 0.2 μmol dNTPs, 0.1 μmol of 10X Buffer IV (Thermo Scientific®) and 0.28 μl of magnesium chloride (concentration 25 mM). Cycling conditions were 5 minutes of pre-incubation at 95°C, followed by 38 cycles of 95°C for one minute, 58°C for 40 seconds, 72°C for 40 seconds, with a final elongation step at 72°C for 10 minutes. Following amplification, a 1.1 μl aliquot of amplified PCR product was added to 9.89 μl of high purity (HiDi) formamide and 0.11 μl of ROX-500 size standard (Applied Biosystems, Warrington, UK). Samples were run on an ×3730 DNA Analyzer and analyzed using GeneMapper 4 software (Applied Biosystems, Warrington UK).

### Data analyses

*Molecular diversity and Population genetics*; exact tests of deviation from Hardy-Weinberg equilibrium (using 10,000 steps in a Markov chain), pairwise *F*_ST_, and AMOVA, were all performed using Arlequin 3.5 [73]. Pairwise *F*_ST_ estimates were used to perform principal co-ordinates analysis in the R-programming environment using routines in the APE package. The *D´* measure of linkage disequilibrium was calculated using the expectation maximization algorithm using LDMax (part of the GOLD software package, freely available at: http://www.sph.umich.edu/csg/abecasis/GOLD/docs/ldmax.html). Haplotypes were inferred using PHASE version 2.1 (1000 iterations, 500 burn-in) [74]. Singletons were removed for haplotype and LD analysis. Haplotype networks were constructed using a median-joining network implemented in Network 4.6.1 and re-colored using Adobe PhotoShop CS4. Nucleotide diversity, tests for departures from neutrality, Fay and Wu’s H test and the HKA test were all performed using DnaSP 5.0 [75]. The chimpanzee *CYP3A5* gene sequence was downloaded from NCBI (http://www.ncbi.nlm.nih.gov/).

*Ecological correlations;* geographic co-ordinates were used to calculate distance from the equator (in kilometers) using the online programme: http://www.movable-type.co.uk/scripts/latlong.html. Raw ecological data for temperature (in degrees Celsius) and precipitation (in mm), at each set of geographic co-ordinates, for 0, 10,000 and 50,000 years ago were extracted from the British Atmospheric Data Centre (http://badc.nerc.ac.uk/home/index.html), from the ALL-5G dataset associated with the Quaternary QUEST (http://researchpages.net/QQ/) [76]. The data were extracted using Python. The raw data have a resolution of 5 degrees latitude and 7.5 degrees longitude and were interpolated to a resolution of 1 degree latitude and 1 degree longitude. The interpolations were done using the smooth.2d function in the fields library of the R-programming environment. An estimate of relative aridity was inferred from extracted temperature and precipitation values corresponding to each geographic location using the de Martonne aridity index [34]. Mantel and partial Mantel tests were performed in the R-programming environment using routines in the APE package [77] and ecodist package [78] respectively.

*Bioinformatics analyses of genetic variation on protein expression and function;* cross-species alignments of *CYP3A5* orthologues (sequences obtained from NCBI: http://www.ncbi.nlm.nih.gov/) were performed using ClustalW software (http://www.ebi.ac.uk/Tools/msa/clustalw2/). Analyses of regulatory motifs in the *CYP3A5* promoter were performed using MatInspector [79], effects of amino acid substitutions on the structure and function of CYP3A5 were performed using PolyPhen2 [80], predictions of mutations which are likely to affect gene splicing were performed using the online Berkeley Drosophila Genome Project splice predictor [81].

*Estimating the age of the clinically relevant CYP3A5 variants*; The gametic phase of CYP3A5 mutations and the –GT microsatellite (rs10536492) was not determined empirically. Allele ages were estimated using data for individuals homozygous for particular haplotypes. As no Ethiopian individual was identified to be a CYP3A5*7 homozygote, this variant could not be dated. Under the stepwise mutation model the variance (ASD) in the microsatellite repeat length, from the most recent common ancestor, is a linear function of the mutation rate (μ) and coalescence time in generations (t); ASD=μt [82,83]. A mutation rate of 4.5×10^-4^ was used to estimate the time to the most recent common ancestor (MRCA) based on average estimates of the mutation rate of dinucleotide microsatellites in the human genome [68,84].

ASD and t were calculated using Ytime software [85]. The microsatellite length of the ancestral MRCA is assumed to be known. For this study the ancestral length of the microsatellite was estimated to be 35; as the majority of CYP3A5*1 haplotypes had 35 repeats. Confidence intervals for the age estimates were obtained from calculating the distances between the ancestral and derived chromosomes under a star-genealogy model; based on the results of network analysis of *CYP3A5* haplotypes. A generation was assumed to be 32 years [46].

## Competing interest

Neil Bradman is Chairman of The Henry Stewart Group and London and City Group of Companies and has extensive business and financial interests including involvement in biotechnology ventures and educational material used by researchers in the life sciences. The research has been funded in part by the London and City Group of Companies and the Melford Charitable Trust of which Neil Bradman is a trustee. The Melford Charitable Trust, London and City Group of Companies and Neil Bradman do have any intellectual, or other, property rights whatsoever with respect to the research which forms the subject matter of the paper. All other authors have no conflict of interest.

## Authors’ contributions

RKB carried out the molecular genetic studies, analyses of data and drafted the manuscript. MK extracted and interpolated all climate data from the British Atmospheric Survey. CAP performed genotyping of the Hypervariable Segment 1 and the Y-chromosome in Ethiopian populations. AT and EB collected all Ethiopian samples which were used for analysis within this study. NNB assisted with the collection of most African samples used in this study, and conceived the initial experimental design of the project. MGT conceived the statistical analyses of the project, in particular those relating to ecological data, and oversaw the writing of the manuscript. All authors read and approved the final manuscript.

## Supplementary Material

Additional file 1 **Table S1.**“The proportion of each inferred *CYP3A5* haplotype observed in each population.” The Table lists the frequencies of each inferred *CYP3A5* haplotype, by population. Click here for file

Additional file 2 **Figure S1.** “The distribution of high-, intermediate- and low- CYP3A5 expression phenotypes, inferred from diplotypes.” The Figure shows inferred CYP3A5 expression phenotypes, assuming that CYP3A5*6 causes low/non-expression of CYP3A5. The size of each circle is proportional to the number of individuals sampled from a given population (see Additional file Table S1). Click here for file

Additional file 3 **Figure S2.** “The distribution of high-, intermediate- and low- CYP3A5 expression phenotypes, inferred from diplotypes.” The Figure shows inferred CYP3A5 expression phenotypes, assuming that CYP3A5*6 does not cause low/non-expression of CYP3A5. The size of each circle is proportional to the number of individuals sampled from a given population (see Additional file Table S1). Click here for file

Additional file 4 **Figures S3a and b.** “Haplotypes inferred from genotype data in 8 populations.” Supplementary Figure 3a shows the composition of each *CYP3A5* haplotype inferred from genotype data for 8 global populations. The frequencies of each haplotype, by population, are shown in Additional file Figure S3b. Click here for file

Additional file 5 **Table S2.** “Geographic co-ordinates, sample size and major language family of each population genotyped in the geographic survey of clinically relevant *CYP3A5* alleles. The *CYP3A5* gene was re-sequenced in five Ethiopian populations.” This Table provides details of all populations which were genotyped, and re-sequenced for this study. Click here for file

Additional file 6 **Table S3.** “A list of the primers used for PCR amplification and sequencing of *CYP3A5*.” Click here for file
