# Supplementary material for: Molecular diversity and population structure at the Cytochrome P450 3A5 gene in Africa
Source: BMC Genet. 2013 May 3;14:34. doi: 10.1186/1471-2156-14-34 (PMC3655848; doi:10.1186/1471-2156-14-34)
Supplement: Additional file 1 — Table S1.“The proportion of each inferred CYP3A5 haplotype observed in each population.” The Table lists the frequencies of each inferred CYP3A5 haplotype, by population. [file 1471-2156-14-34-S1.pdf]

Supplementary Table 1: The proportion of each inferred *CYP3A5* haplotype observed in each population.

Geographic region	Language family	Sample set	Frequencies of inferred haplotypes				
			AG- (*1)	AGinsT (*7)	AA- (*6)	GG- (*3)	GA- (*3/*6)
**Europe**	Indo-European	Southern Armenians	0.05	-	-	0.95	-
	Altaic	Anatolian Turks	0.09	-	-	0.91	-
**Arabian Peninsula**	Afro-Asiatic	Yemeni from Hadramaut	0.12	0.01	0.02	0.84	0.006
	Afro-Asiatic	Yemeni from Sena and Msila	0.27	0.03	0.12	0.58	-
**North Africa**	Afro-Asiatic	Northern Algerians	0.14	0.01	0.05	0.81	-
	Afro-Asiatic	Berbers	0.15	0.01	0.04	0.80	-
	Afro-Asiatic	Northern Sudanese	0.27	0.003	0.11	0.62	-
	Afro-Asiatic	Sudanese from Kordofan	0.33	0.02	0.20	0.45	-
**East Africa**	Afro-Asiatic	Afar	0.17	-	0.18	0.65	-
	Afro-Asiatic	Amhara	0.20	-	0.13	0.65	-
	Nilo-Saharan	Anuak	0.45	0.007	0.26	0.29	-
	Afro-Asiatic	Maale	0.36	0.007	0.14	0.49	0.007
	Afro-Asiatic	Oromo	0.22	-	0.13	0.64	0.007
	Nilo-Saharan	Southern Sudanese	0.40	0.03	0.33	0.24	-
	Niger-Congo B	Chagga	0.51	0.09	0.14	0.26	-
	Niger-Congo B	Bantu speakers from Ssese	0.54	0.21	0.22	0.04	-
**West Africa**	Niger-Congo A	Asante	0.60	0.07	0.22	0.10	-
	Niger-Congo A	Bulsa	0.52	0.12	0.16	0.20	-
	Niger-Congo A	Kasena	0.48	0.13	0.17	0.22	-
	Niger-Congo A	Manjak	0.49	0.07	0.23	0.20	0.01
	Niger-Congo A	Wolof	0.47	0.09	0.18	0.25	-
**West Central Africa**	Afro-Asiatic	Kotoko	0.46	0.05	0.22	0.27	-
	Afro-Asiatic	Shewa Arabs	0.33	0.07	0.21	0.39	0.007
	Niger-Congo A	Mayo Darle	0.40	0.07	0.25	0.28	-
	Niger-Congo A	Mambila from Somie	0.49	0.23	0.18	0.10	-
	Niger-Congo B	Congolese from Brazzaville	0.59	0.09	0.12	0.20	-
	Niger-Congo A	Igbo	0.59	0.09	0.18	0.13	-
**South East Africa**	Niger-Congo B	Chewa	0.54	0.18	0.15	0.13	-
	Niger-Congo B	Lomwe	0.50	0.11	0.22	0.17	-
	Niger-Congo B	Ngoni	0.50	0.11	0.22	0.17	-
	Niger-Congo B	Tumbuka	0.50	0.14	0.22	0.15	-
	Niger-Congo B	Yao	0.61	0.18	0.13	0.09	-
	Niger-Congo B	Sena	0.46	0.17	0.21	0.16	0.006
	Niger-Congo B	Bantu speakers	0.45	0.10	0.19	0.26	-
	Niger-Congo B	Lemba	0.48	0.15	0.24	0.13	-
	Niger-Congo B	Zimbabweans from Mposi	0.45	0.22	0.17	0.16	-
