# Supplementary material for: Molecular diversity and population structure at the Cytochrome P450 3A5 gene in Africa
Source: BMC Genet. 2013 May 3;14:34. doi: 10.1186/1471-2156-14-34 (PMC3655848; doi:10.1186/1471-2156-14-34)

Supplementary Figure 1: The distribution of high-, intermediate- and low- CYP3A5 expression phenotypes, inferred from diplotypes. Expression phenotypes have been inferred assuming that *CYP3A5*6* causes low/non-expression of CYP3A5. The size of each circle is proportional to the number of individuals sampled from a given population (see Supplementary Table 1).

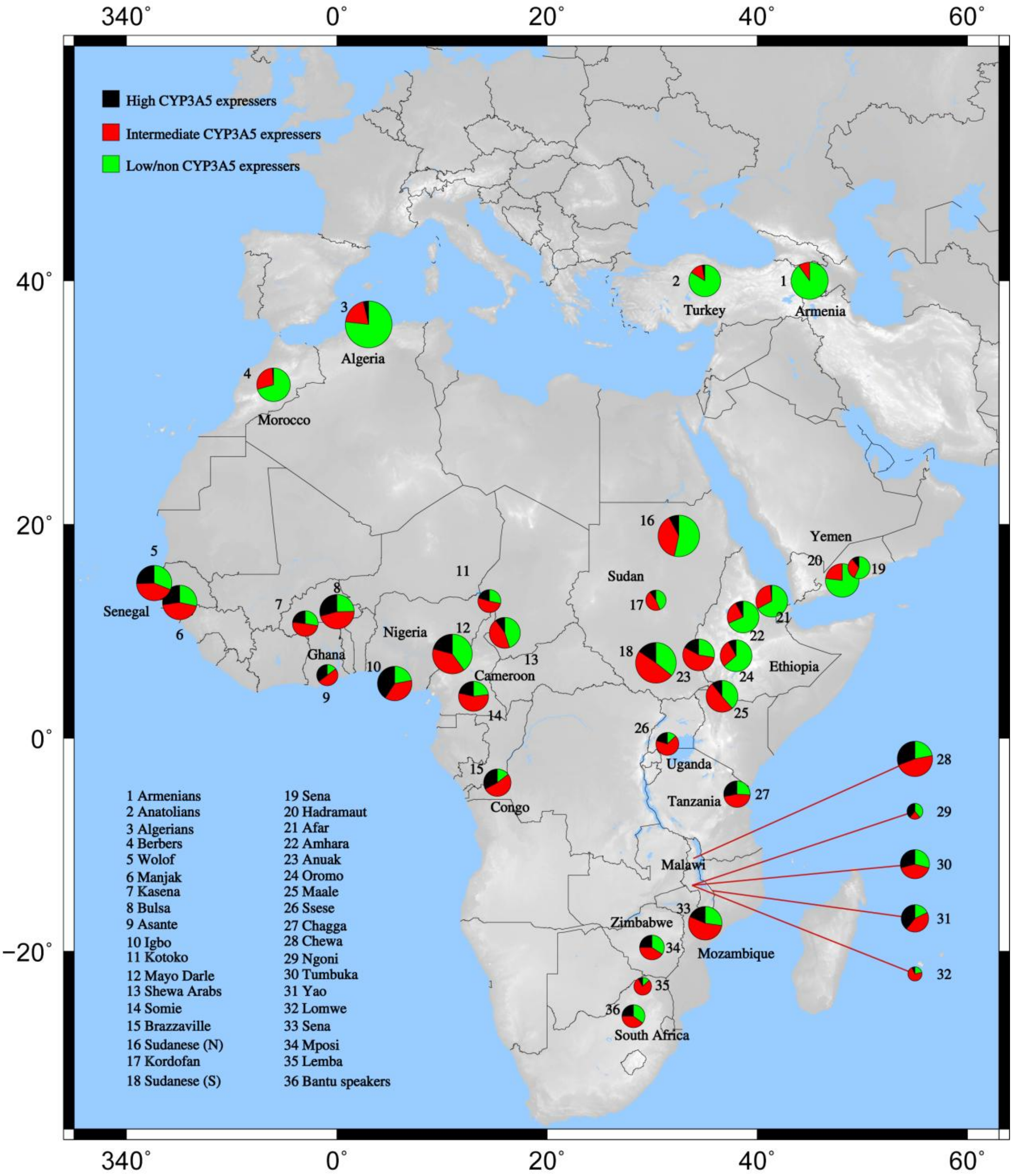

Supplement: Additional file 2 — Figure S1. “The distribution of high-, intermediate- and low- CYP3A5 expression phenotypes, inferred from diplotypes.” The Figure shows inferred CYP3A5 expression phenotypes, assuming that CYP3A5*6 causes low/non-expression of CYP3A5. The size of each circle is proportional to the number of individuals sampled from a given population (see Additional file Table S1). [file 1471-2156-14-34-S2.pdf]
