# Supplementary material for: Molecular diversity and population structure at the Cytochrome P450 3A5 gene in Africa
Source: BMC Genet. 2013 May 3;14:34. doi: 10.1186/1471-2156-14-34 (PMC3655848; doi:10.1186/1471-2156-14-34)

**Supplementary Figure 3a**: Haplotypes inferred from genotype data in 8 populations. The positions of polymorphic sites (numbered from the ATG start codon where base A is +1) are shown along the top. The codes correspond to those used in Figure 5 and *n* is the frequency of each haplotype across the eight populations. Haplogroup refers to whether each haplotype belongs to the *CYP3A5*1, CYP3A5*3, CYP3A5*6, CYP3A5*7* or *CYP3A5*3/*6* cluster of haplotypes. Ancestral alleles, as inferred from the chimpanzee *CYP3A5* allele, at each nucleotide position are coloured in yellow and derived alleles in blue.

| Code | *n* | -795 | -748 | -709 | -705 | -551 | -86 | -74 | -15 | 127 | 182 | 5209 | 5229 | 5244 | 5416 | 6980 | 7201 | 7354 | 13167 | 13370 | 14684 | 14714 | 14877 | 15782 | 15936 | 16785 | 17017 | 17020 | 17112 | 17157 | 18995 | 19159 | 19203 | 26943 | 27044 | 27128 | 27201 | 27520 | 29872 | 30016 | 31493 | 31545 | 31605 | 31812 | Haplogroup |
|---|---|---|---|---|---|---|---|---|---|---|---|---|---|---|---|---|---|---|---|---|---|---|---|---|---|---|---|---|---|---|---|---|---|---|---|---|---|---|---|---|---|---|---|---|---|
| 36a | 3 | T | C | T | C | C | G | C | A | G | C | C | G | C | C | G | C | T | T | G | A | A | A | T | C | G | C | C | C | G | C | G | T | G | A | - | A | C | G | A | T | T | C | G | *3/*6 |
| 36b | 3 | T | C | T | C | C | G | C | A | G | C | C | G | C | C | G | C | T | T | G | A | A | A | T | C | G | C | C | C | G | C | G | T | G | A | - | A | C | G | A | T | T | T | G | *3/*6 |
| 1a | 32 | T | C | T | C | C | G | C | A | G | C | C | G | C | C | A | C | T | T | G | G | A | A | T | C | G | C | C | C | G | C | G | T | G | A | - | A | C | G | A | T | T | C | G | *1 |
| 1aa | 4 | T | C | T | C | A | G | C | A | G | C | C | G | C | C | A | C | T | T | G | G | A | G | T | C | G | C | C | C | G | C | G | T | G | A | - | A | C | G | A | T | T | T | G | *1 |
| 1b | 25 | T | C | T | C | C | G | C | A | G | C | C | G | C | C | A | C | T | T | G | G | A | A | T | C | G | C | C | C | G | C | G | T | G | A | - | A | C | G | A | T | T | T | G | *1 |
| 1bb | 15 | T | C | T | C | A | G | C | A | G | C | C | G | C | C | A | C | T | T | G | G | A | G | T | C | G | C | C | C | G | C | G | T | G | A | - | A | C | G | - | T | T | T | G | *1 |
| 1c | 2 | T | C | T | C | C | G | C | A | G | C | C | G | C | C | A | C | T | T | G | G | A | A | T | C | G | C | C | C | G | C | G | T | G | A | - | A | C | G | - | T | T | C | G | *1 |
| 1cc | 6 | T | C | T | C | A | G | C | A | G | C | C | G | C | C | A | T | T | T | G | G | A | G | T | C | G | C | C | C | G | C | G | T | G | A | - | A | C | G | - | T | T | T | G | *1 |
| 1d | 2 | T | C | T | C | C | G | C | A | G | C | C | G | C | C | A | C | T | T | G | G | A | A | T | C | G | C | C | C | G | C | G | T | G | A | - | A | T | G | A | T | T | C | G | *1 |
| 1dd | 1 | T | C | T | C | A | G | C | A | A | C | C | G | C | C | A | C | T | T | G | G | A | G | T | C | G | C | C | C | G | C | G | T | G | A | - | A | C | G | - | T | T | T | G | *1 |
| 1e | 1 | T | C | T | C | C | G | C | A | G | C | C | G | C | C | A | C | T | T | G | G | A | A | T | C | G | C | C | C | G | C | G | T | G | A | - | A | T | G | - | T | T | C | G | *1 |
| 1ee | 1 | T | C | T | C | A | G | C | A | G | C | C | G | C | C | A | T | T | T | G | G | A | A | T | C | G | C | C | C | G | C | G | T | G | A | - | A | C | G | - | T | T | T | G | *1 |
| 1f | 1 | T | C | T | C | C | G | C | A | G | C | C | G | C | C | A | C | T | T | G | G | A | A | T | C | G | C | C | C | G | C | G | T | G | A | - | A | T | G | - | T | T | T | G | *1 |
| 1ff | 1 | T | C | T | C | A | G | C | A | G | C | C | G | C | C | A | C | T | T | G | G | A | G | T | C | G | C | C | C | G | C | G | T | G | A | - | A | C | G | - | T | T | C | G | *1 |
| 1g | 22 | T | C | T | C | C | G | C | A | G | C | C | G | C | C | A | C | T | T | G | G | A | A | T | C | G | C | C | C | G | T | G | T | G | A | - | A | C | G | A | T | T | C | G | *1 |
| 1gg | 11 | T | C | T | - | C | G | C | A | G | C | C | G | C | C | A | C | T | T | G | G | A | A | T | C | G | C | C | C | G | C | G | T | G | A | - | A | C | G | A | T | T | C | G | *1 |
| 1h | 1 | T | C | T | C | C | G | C | A | G | C | C | G | C | C | A | C | T | T | G | G | A | A | T | C | G | C | C | C | G | T | G | T | G | A | - | A | T | G | A | T | T | C | G | *1 |
| 1hh | 1 | T | C | C | C | C | G | C | A | G | C | C | G | C | C | A | C | T | T | G | G | A | A | T | C | G | C | C | C | G | C | G | T | G | A | - | A | C | G | A | T | T | C | G | *1 |
| 1i | 1 | T | C | T | C | C | G | C | A | G | C | C | G | C | C | A | C | T | T | G | G | A | A | T | C | G | C | C | C | T | C | G | T | G | A | - | A | C | G | A | T | T | C | G | *1 |
| 1ii | 13 | T | G | T | C | C | G | C | A | G | C | C | G | C | C | A | C | T | T | G | G | A | A | T | C | G | C | C | C | G | C | G | T | G | A | - | A | C | G | A | T | T | C | G | *1 |
| 1j | 2 | T | C | T | C | C | G | C | A | G | C | C | G | C | C | A | C | T | T | G | G | A | A | T | C | A | C | C | C | G | C | G | T | G | A | - | A | C | G | - | T | T | C | G | *1 |
| 1jj | 20 | A | C | T | C | C | G | C | A | G | C | C | G | C | C | A | C | T | T | G | G | A | A | T | C | G | C | C | C | T | C | G | T | G | A | - | A | C | G | A | T | T | C | G | *1 |
| 1k | 26 | T | C | T | C | C | G | C | A | G | C | C | G | C | C | A | C | T | T | G | G | A | A | C | C | G | C | C | C | G | C | G | T | G | A | - | A | C | G | A | T | T | C | G | *1 |

| | | | | | | | | | | | | | | | | | | | | | | | | | | | | | | | | | | | | | | | | | | | | | |
|---|---|---|---|---|---|---|---|---|---|---|---|---|---|---|---|---|---|---|---|---|---|---|---|---|---|---|---|---|---|---|---|---|---|---|---|---|---|---|---|---|---|---|---|---|---|
| **1kk** | 2 | A | C | T | C | C | G | C | A | G | C | C | G | C | C | A | T | T | T | G | G | A | A | T | C | G | C | C | C | T | C | G | T | G | A | - | A | C | G | A | C | T | C | G | *1 |
| **1l** | 2 | T | C | T | C | C | G | C | A | G | C | C | G | C | C | A | C | T | T | G | G | A | G | T | C | G | C | C | C | G | C | G | T | G | A | - | A | C | G | - | T | T | T | G | *1 |
| **1m** | 1 | T | C | T | C | C | G | C | A | G | C | C | G | C | C | A | C | T | T | G | G | G | A | T | C | G | C | C | C | G | C | G | T | G | A | - | A | C | G | - | T | T | C | G | *1 |
| **1n** | 1 | T | C | T | C | C | G | C | A | G | C | C | G | C | C | A | C | T | T | A | G | A | A | T | C | G | C | C | C | G | C | G | T | G | A | - | A | C | G | A | T | T | C | G | *1 |
| **1o** | 10 | T | C | T | C | C | G | C | A | G | C | C | G | C | C | A | C | T | C | G | G | A | A | T | C | G | C | C | C | G | C | G | T | G | A | - | A | C | G | A | T | T | T | G | *1 |
| **1p** | 1 | T | C | T | C | C | G | C | A | G | C | C | G | C | C | A | T | T | T | G | G | G | A | T | C | G | C | C | C | G | C | G | T | G | A | - | A | C | G | A | T | T | C | G | *1 |
| **1q** | 1 | T | C | T | C | C | G | C | A | G | C | C | G | C | C | A | T | T | T | G | G | A | A | T | C | G | C | C | C | G | C | G | T | G | A | - | A | C | G | A | T | T | C | G | *1 |
| **1r** | 2 | T | C | T | C | C | G | C | A | G | C | C | A | C | C | A | C | T | T | G | G | A | A | T | C | G | C | C | C | G | T | G | T | G | A | - | A | C | G | A | T | T | C | G | *1 |
| **1s** | 22 | T | C | T | C | C | G | C | A | G | C | T | G | C | C | A | C | T | T | G | G | A | A | T | C | G | C | C | C | G | C | G | T | G | A | - | A | C | G | A | T | T | C | G | *1 |
| **1t** | 1 | T | C | T | C | C | G | C | A | G | A | C | G | C | C | A | C | T | T | G | G | A | A | C | C | G | C | C | C | G | C | G | T | G | A | - | A | C | G | A | T | T | C | G | *1 |
| **1u** | 1 | T | C | T | C | C | G | C | A | G | A | C | G | C | C | A | C | T | T | G | G | A | A | T | C | G | C | C | C | G | C | G | T | G | A | - | A | C | G | A | T | T | C | G | *1 |
| **1v** | 2 | T | C | T | C | C | G | C | A | A | C | C | G | C | C | A | C | T | T | G | G | A | A | T | A | G | C | C | C | G | C | G | T | G | A | - | A | C | T | A | T | T | C | G | *1 |
| **1w** | 1 | T | C | T | C | C | G | C | C | G | C | C | G | C | C | A | C | T | T | G | G | A | A | T | C | G | C | C | C | G | C | G | T | G | A | - | A | C | G | - | T | T | T | G | *1 |
| **1x** | 2 | T | C | T | C | A | G | C | A | G | C | C | G | C | C | A | C | T | T | G | G | A | A | T | C | G | C | C | C | G | C | G | T | G | A | - | A | C | G | A | T | T | C | G | *1 |
| **1y** | 1 | T | C | T | C | A | G | C | A | G | C | C | G | C | C | A | C | C | T | G | G | A | A | T | C | G | C | C | C | G | C | G | T | G | A | - | A | C | G | A | T | T | C | G | *1 |
| **1z** | 7 | T | C | T | C | A | G | C | A | G | C | C | G | C | C | A | C | T | T | G | G | A | A | T | C | G | C | C | C | G | C | G | T | G | A | - | A | C | G | - | T | T | T | G | *1 |
| **3a** | 1 | T | C | T | C | C | G | C | A | G | C | C | G | C | C | G | C | T | T | G | G | A | A | T | C | A | C | C | C | G | C | G | T | G | A | - | A | C | G | A | T | T | T | G | *3 |
| **3aa** | 2 | A | C | T | C | C | G | C | A | G | C | C | G | C | C | G | C | T | T | G | G | A | A | T | C | G | C | C | C | T | C | G | T | G | A | - | A | C | G | A | T | T | C | G | *3 |
| **3b** | 377 | T | C | T | C | C | G | C | A | G | C | C | G | C | C | G | C | T | T | G | G | A | A | T | C | G | C | C | C | G | C | G | T | G | A | - | A | C | G | A | T | T | T | G | *3 |
| **3bb** | 2 | T | C | T | C | C | G | C | A | G | C | C | G | C | C | G | C | T | T | G | G | A | A | C | C | G | C | C | C | G | C | G | T | G | A | - | A | C | G | A | T | T | C | G | *3 |
| **3c** | 2 | T | C | T | C | C | G | C | A | G | C | C | G | C | C | G | C | T | T | G | G | A | A | T | C | G | C | C | C | G | C | G | T | G | A | - | A | T | G | A | T | T | T | G | *3 |
| **3d** | 1 | T | C | T | C | C | G | C | A | G | C | C | G | C | C | G | C | T | T | G | G | A | A | T | C | G | C | C | C | G | C | G | T | A | A | - | A | C | G | A | C | T | T | G | *3 |
| **3e** | 15 | T | C | T | C | C | G | T | A | G | C | C | G | C | C | G | C | T | T | G | G | A | A | T | C | G | C | C | C | G | C | G | T | G | A | - | A | C | G | A | T | T | T | G | *3 |
| **3f** | 1 | T | C | T | C | C | G | C | A | G | C | C | G | C | C | G | C | T | T | G | G | A | A | T | C | G | C | C | C | G | T | G | T | G | G | - | A | C | G | - | T | T | T | G | *3 |
| **3g** | 3 | T | C | T | C | C | G | C | A | G | C | C | G | C | C | G | C | T | T | G | G | A | A | T | C | G | C | C | C | G | C | G | T | G | A | - | A | C | G | - | T | T | T | G | *3 |
| **3h** | 1 | T | C | T | C | C | G | C | A | G | C | C | G | C | C | G | C | T | T | G | G | A | A | T | C | G | C | C | C | G | T | G | T | G | A | - | A | C | G | - | T | T | T | G | *3 |
| **3i** | 4 | T | C | T | C | C | G | C | A | G | C | C | G | C | C | G | C | T | T | G | G | A | A | T | C | G | C | C | C | G | C | G | T | G | G | - | A | C | G | A | T | T | T | G | *3 |
| **3j** | 1 | T | C | T | C | C | G | C | A | G | C | C | G | C | C | G | C | T | T | G | G | A | A | T | C | G | C | C | C | G | C | G | T | G | A | - | A | C | G | - | T | C | T | G | *3 |
| **3k** | 1 | T | C | T | C | C | G | C | A | G | C | T | G | C | C | G | C | T | T | G | G | A | A | T | C | G | C | T | C | G | C | G | T | G | A | - | A | T | G | A | T | T | T | G | *3 |

| | | | | | | | | | | | | | | | | | | | | | | | | | | | | | | | | | | | | | | | | | | | | | |
|---|---|---|---|---|---|---|---|---|---|---|---|---|---|---|---|---|---|---|---|---|---|---|---|---|---|---|---|---|---|---|---|---|---|---|---|---|---|---|---|---|---|---|---|---|---|
| **3l** | 1 | T | C | T | C | C | G | C | A | G | C | C | G | C | C | G | C | T | T | G | G | A | A | T | C | G | C | C | C | G | C | G | T | G | A | - | A | C | G | A | T | C | T | G | ***3** |
| **3m** | 1 | T | C | T | C | C | G | C | A | G | C | C | G | C | C | G | C | T | T | G | G | A | A | T | C | G | C | C | C | G | C | A | T | G | A | - | A | C | G | A | T | T | T | G | ***3** |
| **3n** | 23 | T | C | T | C | C | G | C | A | G | C | T | G | C | C | G | C | T | T | G | G | A | A | T | C | G | C | C | C | G | C | G | T | G | A | - | A | C | G | A | T | T | T | G | ***3** |
| **3o** | 1 | T | C | T | C | C | A | C | A | G | C | C | G | C | C | G | C | T | T | G | G | A | A | T | C | G | C | C | C | G | C | G | T | G | A | - | A | C | G | A | T | T | T | G | ***3** |
| **3p** | 1 | T | C | T | C | C | G | T | A | G | C | C | G | C | C | G | C | T | T | G | G | A | A | T | C | G | C | C | C | G | C | G | T | G | A | - | G | C | G | A | T | T | T | G | ***3** |
| **3q** | 1 | T | C | T | C | C | G | C | A | G | C | T | G | C | C | G | C | T | T | G | G | A | A | T | C | G | C | C | C | G | C | G | T | G | A | - | A | T | G | A | T | T | T | G | ***3** |
| **3r** | 51 | T | C | T | C | C | G | C | A | G | C | C | G | C | C | G | C | T | T | G | G | A | A | T | C | G | C | C | C | G | C | G | T | G | A | - | A | C | G | A | C | T | T | G | ***3** |
| **3s** | 1 | T | C | T | C | C | G | C | A | G | C | C | G | C | C | G | C | T | T | G | G | A | A | T | C | G | C | C | C | G | C | G | T | G | A | - | A | C | G | A | T | T | T | A | ***3** |
| **3t** | 2 | T | C | T | C | C | G | C | A | G | C | T | G | C | C | G | C | T | T | G | G | A | A | T | C | G | C | C | T | G | C | G | T | G | A | - | A | C | G | A | T | T | T | G | ***3** |
| **3u** | 2 | T | C | T | C | C | G | C | A | G | C | C | G | C | T | G | C | T | T | G | G | A | A | T | C | G | C | C | C | G | C | G | T | G | A | - | A | C | G | A | T | T | T | G | ***3** |
| **3v** | 2 | T | C | T | C | C | G | C | A | G | C | C | G | T | C | G | C | T | T | G | G | A | A | T | C | G | C | C | C | G | C | G | T | G | A | - | A | C | G | A | T | T | T | G | ***3** |
| **3w** | 1 | A | C | T | C | C | G | C | A | G | C | T | G | C | C | G | C | T | T | G | G | A | A | T | C | G | C | C | C | G | C | G | T | G | A | - | A | C | G | A | T | T | T | G | ***3** |
| **3x** | 1 | T | G | T | C | C | G | C | A | G | C | C | G | C | C | G | C | T | T | G | G | A | A | T | C | G | C | C | C | G | C | G | T | G | A | - | A | C | G | A | T | T | T | G | ***3** |
| **3y** | 9 | T | C | T | C | C | G | C | A | G | C | C | G | C | C | G | C | T | T | G | G | A | A | T | C | G | C | C | C | G | C | G | T | G | A | - | A | C | G | A | T | T | C | G | ***3** |
| **3z** | 1 | T | G | T | C | C | G | C | A | G | C | C | G | C | C | G | C | T | T | G | G | A | A | T | C | G | C | C | C | G | C | G | T | G | A | - | A | C | G | A | T | T | C | G | ***3** |
| **6a** | 122 | T | C | T | C | C | G | C | A | G | C | C | G | C | C | A | C | T | T | G | A | A | A | T | C | G | C | C | C | G | C | G | T | G | A | - | A | C | G | A | T | T | C | G | ***6** |
| **6b** | 1 | T | C | T | C | C | G | C | A | G | C | C | G | C | C | A | C | T | T | G | A | A | A | T | C | G | C | C | C | G | C | G | T | G | A | - | A | T | G | A | T | T | C | G | ***6** |
| **6c** | 2 | T | C | T | C | C | G | C | A | G | C | C | G | C | C | A | C | T | T | G | A | A | A | T | C | G | C | C | C | G | C | G | T | G | A | - | A | C | G | - | T | T | C | G | ***6** |
| **6d** | 3 | T | C | T | C | C | G | C | A | G | C | C | G | C | C | A | C | T | T | G | A | A | A | T | C | G | C | C | C | G | C | G | C | G | A | - | A | C | G | A | T | T | C | G | ***6** |
| **6e** | 1 | T | C | T | C | C | G | C | A | G | C | C | G | C | C | A | C | T | T | G | A | A | A | T | C | G | T | C | C | G | C | G | T | G | A | - | A | C | G | A | T | T | C | G | ***6** |
| **6f** | 2 | T | C | T | C | C | G | C | A | G | C | C | G | C | C | A | T | T | T | G | A | A | A | T | C | G | C | C | C | G | C | G | T | G | A | - | A | C | G | A | T | T | C | G | ***6** |
| **6g** | 1 | T | C | T | C | A | G | C | A | G | C | C | G | C | C | A | C | T | T | G | A | A | A | T | C | G | C | C | C | G | C | G | T | G | A | - | A | C | G | - | T | T | T | G | ***6** |
| **7a** | 2 | T | C | T | C | C | G | C | A | G | C | C | G | C | C | A | C | T | T | G | G | A | A | T | C | G | C | C | C | G | C | G | T | G | A | T | A | C | G | A | T | T | C | G | ***7** |

**Supplementary Figure 3b:** The frequencies of each haplotype (shown in Supplementary Figure 3a) by population.

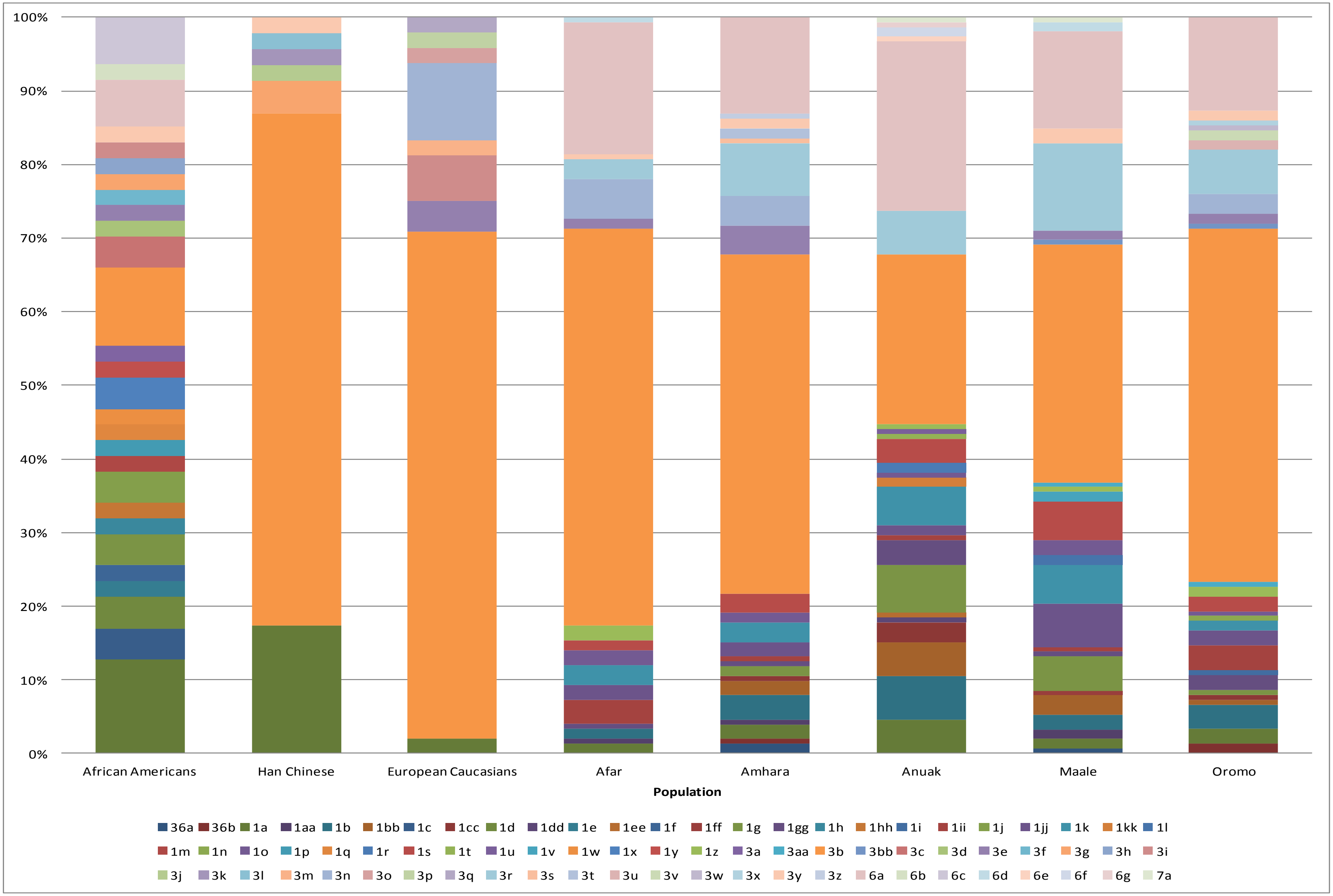

Supplement: Additional file 4 — Figures S3a and b. “Haplotypes inferred from genotype data in 8 populations.” Supplementary Figure 3a shows the composition of each CYP3A5 haplotype inferred from genotype data for 8 global populations. The frequencies of each haplotype, by population, are shown in Additional file Figure S3b. [file 1471-2156-14-34-S4.pdf]
