# Supplementary material for: Molecular diversity and population structure at the Cytochrome P450 3A5 gene in Africa
Source: BMC Genet. 2013 May 3;14:34. doi: 10.1186/1471-2156-14-34 (PMC3655848; doi:10.1186/1471-2156-14-34)
Supplement: Additional file 5 — Table S2. “Geographic co-ordinates, sample size and major language family of each population genotyped in the geographic survey of clinically relevant CYP3A5 alleles. The CYP3A5 gene was re-sequenced in five Ethiopian populations.” This Table provides details of all populations which were genotyped, and re-sequenced for this study. [file 1471-2156-14-34-S5.pdf]

**Supplementary Table 2:** Geographic co-ordinates, sample size and major language family of each population genotyped in the geographic survey of clinically relevant *CYP3A5* alleles. The *CYP3A5* gene was re-sequenced in five Ethiopian populations

| Geographic region | Country | Sample set | Major language family | Latitude | Longitude | Number of individuals |
|---|---|---|---|---|---|---|
| Europe | Armenia | Southern Armenians (*b*) | Indo-European | 40.00 | 45.00 | 100 |
| | Turkey | Anatolian Turks (*a*) | Altaic | 39.00 | 35.00 | 74 |
| Arabian Peninsula | Yemen | Yemeni from Hadramaut (*b*) | Afro-Asiatic | 14.91 | 48.07 | 82 |
| | | Yemeni from Sena and Msila (*b*) | Afro-Asiatic | 16.08 | 49.67 | 37 |
| North Africa | Algeria | Northern Algerians (*b*) | Afro-Asiatic | 35.505 | -1.045 | 161 |
| | Morocco | Berbers (*a*) | Afro-Asiatic | 34.03 | -6.84 | 86 |
| | Sudan | Northern Sudanese (*b*) | Afro-Asiatic | 15.59 | 32.52 | 136 |
| | | Sudanese from Kordofan (*b*) | Afro-Asiatic | 13.08 | 30.35 | 30 |
| East Africa | Ethiopia | Afar (*a*) | Afro-Asiatic | 11.602 | 41.360 | 73 |
| | | Amhara (*a*) | Afro-Asiatic | 9.869 | 38.660 | 76 |
| | | Anuak (*a*) | Nilo-Saharan | 7.953 | 34.412 | 76 |
| | | Maale (*a*) | Afro-Asiatic | 5.715 | 36.643 | 75 |
| | | Oromo (*a*) | Afro-Asiatic | 7.837 | 37.308 | 74 |
| | Sudan | Southern Sudanese (*b*) | Nilo-Saharan | 5.18 | 31.77 | 125 |
| | Tanzania | Chagga (*a*) | Niger-Congo B | -5.38 | 38.05 | 50 |
| | Uganda | Bantu speakers from Ssese (*b*) | Niger-Congo B | -0.57 | 31.45 | 39 |
| West Africa | Ghana | Asante (*a*) | Niger-Congo A | 5.82 | -2.82 | 35 |
| | | Bulsa (*a*) | Niger-Congo A | 10.73 | -1.29 | 90 |
| | | Kasena (*a*) | Niger-Congo A | 10.89 | -1.09 | 47 |
| | Senegal | Manjak (*a*) | Niger-Congo A | 12.986 | -15.88 | 94 |
| | | Wolof (*a*) | Niger-Congo A | 14.687 | -17.453 | 94 |
| West Central Africa | Cameroon | Kotoko (*a*) | Afro-Asiatic | 13.00 | 14.5 | 40 |
| | | Shewa Arabs (*a*) | Afro-Asiatic | 15.05 | 12.11 | 69 |
| | | Cameroonians from Mayo Darle (*b*) | Niger-Congo A | 6.47 | 11.55 | 118 |
| | | Mambila from Somie, in the Cameroonian Grassfields (*b*) | Niger-Congo A | 6.00 | 12.5 | 65 |
| | Congo | Congolese from Brazzaville (*b*) | Niger-Congo B | -4.26 | 15.28 | 55 |
| | Nigeria | Igbo (*a*) | Niger-Congo A | 4.95 | 8.32 | 88 |
| South East Africa | Malawi | Chewa (*a*) | Niger-Congo B | -13.47 | 34.188 | 92 |
| | | Lomwe (*a*) | Niger-Congo B | -13.47 | 34.188 | 18 |
| | | Ngoni (*a*) | Niger-Congo B | -13.47 | 34.188 | 18 |
| | | Tumbuka (*a*) | Niger-Congo B | -13.47 | 34.188 | 62 |
| | | Yao (*a*) | Niger-Congo B | -13.47 | 34.188 | 56 |
| | Mozambique | Bantu speakers from Sena (*b*) | Niger-Congo B | -17.44 | 35.05 | 85 |
| | South Africa | Bantu speakers from Pretoria (*b*) | Niger-Congo B | -25.71 | 28.23 | 41 |
| | Zimbabwe | Lemba (*a*) | Niger-Congo B | -23.095 | 29.075 | 24 |
| | | Zimbabweans from Mposi (*b*) | Niger-Congo B | -19.67 | 30.00 | 52 |
