# Supplementary material for: Molecular diversity and population structure at the Cytochrome P450 3A5 gene in Africa
Source: BMC Genet. 2013 May 3;14:34. doi: 10.1186/1471-2156-14-34 (PMC3655848; doi:10.1186/1471-2156-14-34)
Supplement: Additional file 6 — Table S3. “A list of the primers used for PCR amplification and sequencing of CYP3A5.” [file 1471-2156-14-34-S6.pdf]

Supplementary Table 3: A list of the primers used for PCR amplification and sequencing of *CYP3A5*.

Region of *CYP3A5*	Primer sequences	Fragment size (base pairs)	Position from the ATG start codon	Position on chromosome 7 (NCBI Build 132)	Annealing temperature (°C)	Number of PCR cycles
**Promoter**	**F:** 5´-ACACATCTTTACCCACGAAATTC-3´<br>**R**: 5´-TTATGAGGAATTAAGTGGCAGAA-3´	520	-914 › -395	99278433 – 99277914	55	40
**Promoter**	**F:** 5´-CGCCACTTTCCTTCTTCAACTG-3´<br>**R**: 5´-TAAGGAAAAATTTTAGCAGAAGGGG-3´	511	-511 › -22	99278030 – 99277541	55	40
**Exon 1**	**F:** 5´-GAACCCAGAACCCTTGGACT-3´<br>**R**: 5´-TCCCACTACCAAATGCTGTCCCT-3´	598	-277 › 320	99277796 – 99277199	59	38
**Exon 2**	**F:** 5´-AGACTTCAGCTGCTTTCAGC-3´<br>**R**: 5´-TGGGCTACCATATCATGCACAGG-3´	595	3462 › 4056	99274057 – 99273463	61	38
**Exon 3**	**F:** 5´-AGCTTCCTTCAACTGCCAGTGAA-3´<br>**R**: 5´-ACCACAACTTTGCACAAAGGCT-3´	594	5180 › 5773	99272339 – 99271746	63	38
**Exon 4**	**F:** 5´-ATGGGCCCCACACCAACTGC-3´<br>**R**: 5´-TACCACTGGGCGGGACAGGAT-3´	715	6747 › 7461	99270772 – 99270058	64	38
**Exons 5 and 6**	**F:** 5´-TACACTCAGAAGAGGCTAGGCA-3´<br>**R**: 5´-CATCTTACCCAATGCAAGGCAA-3´	1226	12444 › 13670	99265075 – 99263849	58	40
**Exon 7**	**F:** 5´-TATGACTGGGCTCCTTGACCT-3´<br>**R**: 5´-TTTGTGGTGGGGTGTTGACAGCT-3´	618	14324 › 14941	99263195 – 99262578	61	38
**Exon 8**	**F:** 5´-GTCGCCGGCCTGAAAGAAGGGC-3´<br>**R**: 5´-ATTCTTTACCAATCTGTGATATGA-3´	651	15641 › 16291	99261878 – 99261228	58	40
**Exon 9**	**F:** 5´-AGATGGAACCGGCAACTCTTT-3´<br>**R**: 5´-CCAAGTAGAGGTTCTCACTTGGTG-3´	691	16708 › 17398	99260811 – 99260121	58	40
**Exon 10**	**F:** 5´-TGGGAAAAAGCCTACCCCAT-3´<br>**R**: 5´-TCTCCTCAGAGGCTTCCTAC-3´	678	18861 › 19538	99258658 – 99257981	55	40
**Exon 11**	**F:** 5´-CCCTGGGGTGAGGATGGTCT-3´<br>**R**: 5´-TGTCTTGTGCTGGGACTGTGGATG-3´	671	26880 › 27550	99250639 – 99249969	61	38
**Exon 12**	**F:** 5´-TCTCATCTCAAGAAACGCTCCT-3´<br>**R**: 5´-CATGTCATGCTAATCTGTGTGGAC-3´	607	29434 › 30040	99248085 – 99247479	55	40
**Exon 13**	**F:** 5´-ACGATGGATGGTGAGTGCTT-3´<br>**R**: 5´-TCTGATGAGAGCTCAGGAGGAGTT-3´	600	31297 › 31876	99246222 – 99245643	58	40
